# Effects of nitrogen deposition on territory numbers of breeding birds

**DOI:** 10.1111/cobi.70114

**Published:** 2025-08-15

**Authors:** Kim Meichtry‐Stier, Pius Korner, Simon Birrer, Peter Knaus

**Affiliations:** ^1^ Swiss Ornithological Institute Sempach Switzerland

**Keywords:** atmospheric nitrogen deposition, biodiversity, bird densities, eutrophication, Europe, Switzerland, biodiversidad, densidad ornitológica, deposición de nitrógeno atmosférico, Europa, Suiza

## Abstract

Deposition of atmospheric N (nitrogen) is assumed to be a major cause of biodiversity decline in Europe. To date, few studies on the direct or indirect effects of N on bird species have been conducted. Using Swiss bird count data and habitat data, we analyzed the correlation of N deposition with numbers of territories of 112 breeding bird species. Fifty‐five species had a negative correlation with N, and 21 had a positive correlation. Thirty‐six species showed no clear linear relationship. Insectivorous and herbivorous species were more negatively associated with N deposition (insectivores: 23 species with well‐supported negative correlation vs. 9 species with well‐supported positive correlation; herbivores: 6 vs. 1) than omnivorous birds or birds feeding on vertebrates (2 with negative correlation vs. 2 with positive correlation and 1 with negative correlation vs. 1 with positive correlation, respectively). Species associated with forest (23 negative vs. 3 positive), human settlement and wetland (each 3 negative vs. 0 positive), and birds that could not be attributed to a single guild (3 negative vs. 1 positive) showed mainly a negative relationship with N deposition, whereas more positive than negative correlations were found for alpine (0 negative vs. 2 positive) and common farmland species (0 negative vs. 7 positive). Ground‐nesting species were more negatively associated with N deposition (8 negative vs. 2 positive) than species that nest high aboveground (24 negative vs. 11 positive). The negative correlation of N deposition with territory numbers was slightly more pronounced in long‐distance migrant species (9 negative vs. 3 positive) than in resident or short‐distance migrants (23 negative vs. 10 positive). Rare species were excluded, likely biasing farmland bird results positively. We assumed that differences in the vegetation due to higher N inputs were the main cause for our results. Reduced plant diversity, altered vegetation structure, and more frequent mowing affect breeding habitat and availability of food (invertebrates and seeds) for birds. In Switzerland, airborne N deposition exceeds by far the critical loads for most ecosystems. Our results highlight the urgent need to reduce N deposition to protect a wide range of Swiss bird species.

## Abstract (es)

Efectos de la deposición de nitrógeno sobre el número de territorios de aves reproductoras

**Resumen:**

La deposición atmosférica de nitrógeno (N) es considerada una de las principales causas del declive de la biodiversidad en Europa. Hasta la fecha, hay pocos estudios sobre los efectos directos o indirectos del N en las especies de aves. Utilizamos datos de censos suizos de aves y datos sobre hábitats, analizamos la correlación entre la deposición de N y el número de territorios de 112 especies de aves reproductoras. Cincuenta y cinco especies mostraron una correlación negativa con el N y 21 mostraron una correlación positiva. Treinta y seis especies no mostraron una relación lineal clara. Las especies insectívoras y herbívoras mostraron una asociación más negativa con la deposición de N (insectívoros: 23 especies con una correlación negativa bien fundamentada vs nueve especies con una correlación positiva bien fundamentada; herbívoros: seis vs una) que las aves omnívoras o las que se alimentan de vertebrados (dos con correlación negativa vs dos con correlación positiva y una con correlación negativa vs una con correlación positiva, respectivamente). Las especies asociadas con los bosques (23 negativas vs tres positivas), asentamientos humanos y humedales (tres negativas vs cero positivas cada una), y las aves que no podían incluirse en solo un gremio (tres negativas vs una positiva) mostraron principalmente una relación negativa con la deposición de nitrógeno, mientras que encontramos más correlaciones positivas que negativas para las especies alpinas (cero negativas vs dos positivas) y las especies comunes de tierras de cultivo (cero negativas vs siete positivas). Las especies que anidan en el suelo mostraron una asociación más negativa con la deposición de nitrógeno (ocho negativas vs dos positivas) que las especies que anidan en altura (24 negativas vs once positivas). La correlación negativa entre la deposición de nitrógeno y el número de territorios fue ligeramente más pronunciada en las especies migratorias de larga distancia (nueve negativas vs tres positivas) que en las residentes o migratorias de corta distancia (23 negativas vs diez positivas). Excluimos las especies raras, lo que probablemente causó un sesgo positivo en los resultados de las aves de tierras agrícolas. Supusimos que las diferencias en la vegetación debidas a mayores aportes de N eran la causa principal de nuestros resultados. La reducción de la diversidad vegetal, la alteración de la estructura de la vegetación y el aumento de la frecuencia de siega afectan al hábitat de cría y a la disponibilidad de alimento (invertebrados y semillas) para las aves. En Suiza, la deposición atmosférica de N supera con creces las cargas críticas para la mayoría de los ecosistemas. Nuestros resultados resaltan la urgente necesidad de reducir la deposición de N para proteger una amplia gama de especies de aves suizas.

## INTRODUCTION

Over the last decades, industrialization, traffic, and especially intensive agriculture have led to high emission of nitrogen (N) into the air. This atmospheric N is carried over large distances and threatens natural and seminatural habitats that originally experienced low N input, such as forests, wetlands, and traditionally used grassland (Rihm & Achermann, 2016). Deposition of atmospheric N and the associated habitat alteration is assumed to be a major cause of the decline of biodiversity in Europe (Wright et al., 2018). In the last 20 years, research on increased N input and its effects on biodiversity has increased, especially regarding plant species richness and composition (Bobbink et al., 2010; Clark & Tilman, 2008; De Schrijver et al., 2011; Humbert et al., 2015; Kleijn et al., 2009; Maskell et al., 2010; Roth et al., 2013; Soons et al., 2017) and the functioning of soil biota (Liu et al., 2021; Treseder, 2008; Wang et al., 2018). Increased N in the environment could increase net primary productivity (Stevens et al., 2015), which in turn may reduce the diversity of terrestrial vegetation by favoring common, fast‐growing species adapted to high nutrient availability (Bobbink et al., 1998; Hautier et al., 2009; Stevens et al., 2006, 2004; Suding et al., 2005; Tilman, 1993). In addition, N may decrease diversity through various factors, such as soil acidification, ammonium toxicity, and increased susceptibility to secondary stress factors, such as pests, drought, and other disturbances (Bobbink et al., 1998; Gilliam, 2006; Tresch et al., 2023; Vitousek et al., 1997). However, less is known about the effects of N on organisms in higher tropic levels, such as insects (Hohl et al., 2021; Pöyry et al., 2017; Roth et al., 2021; Vogels et al., 2017) and birds (Billeter et al., 2008; Ewing et al., 2020; Stevens et al., 2018; van den Burg, 2021). Scheibler (2015) found a negative effect of high N deposition on wood warbler *Phylloscopus sibilatrix* occurrence.

In Switzerland, the highest N emissions occur in the lowlands and hilly zones of the Swiss Plateau and the Canton Tessin, where intense farming with regionally high livestock numbers, heavy traffic, and industry is found. Emissions are lower in the mountainous regions of the Jura and Alps (Rihm & Achermann, 2016). In Switzerland, airborne N deposition exceeds by far the maximal amount of N per hectare per year below which no harmful effects on biodiversity would be expected (critical loads [Rihm & Achermann, 2016]). In the Swiss Bird Atlas (Knaus et al., 2018), a correlation graph shows that the higher the N deposition, the lower the bird species richness. In light of these facts and the ongoing biodiversity crisis (Almond et al., 2022; Brondízio et al., 2019), we believe it to be important to learn more about the effects of N on bird species. To our knowledge, we are the first to study correlations between aerial N deposition and bird territory numbers.

Specifically, we investigated whether and to what extend different ecological guilds might be correlated with N deposition. We assumed birds breeding on or near the ground to be negatively affected by N because higher N input leads to faster and denser plant growth, which could impede nesting attempts. Also, faster and denser growing vegetation results in earlier mowing dates of meadows and thus causes nest losses in ground‐breeding species. We further expected high N inputs to have different effects on birds in different food guilds. Nitrogen input by itself and changes in vegetation could alter insect richness and occurrence (Roth et al., 2021), which in turn could change food availability and timing of food availability for invertebrate‐feeding species. Similarly, because most long‐distance migrants forage on invertebrates, such species may show a negative correlation with N input, whereas resident and short‐distance migrant birds often have a mixed or plant‐based diet and may be less negatively affected by N deposition.

## METHODS

### Bird and habitat data

Bird data were collected within the frameworks of the Swiss Breeding Bird Atlas (Knaus et al., 2018) and the Swiss Biodiversity Monitoring (Koordinationsstelle BDM, 2014) from 2012 to 2016. In 2318 1×1‐km survey areas, volunteers mapped all bird observations recorded during the breeding season. Censuses were conducted 3 times (2 times in survey areas above the forest line) from mid‐April to mid‐July. Observations from the 3 censuses were aggregated to territories based on behavioral criteria, such as singing. We restricted our analyses to 112 species found in at least 50 survey areas (Appendix S1). To reduce the number of zeros and exclude areas where a species might not exist for reasons other than the factors we examined (e.g., species’ native range covers only the southern parts of Switzerland), only survey areas within 10 km of another survey area where a species was found were included in the analyses. For species absent from large areas due to reasons linked to high N inputs, we therefore likely underestimated the N effect to some degree.

In the context of the Swiss breeding bird atlas, habitat variables were compiled for each survey area. We took habitat data from the Arealstatistik and Topografisches Landschaftsmodell and the VECTOR25 data from the federal Office of Topography (Bundesamt für Landestopografie, 2007) and data on topographic variables from the digital elevation model of the Swiss federal Statistical Office (Bundesamt für Landestopografie, 2005; Bundesamt für Statistik [BfS], 2014, 2015).

Data on N deposition were calculated by the company Meteotest on behalf of the Swiss federal Office for the Environment (BAFU) for the year 2015 (Rihm & Achermann, 2016). Nitrogen deposition was not directly measured at the studied sites, but we estimated it with an approach that combined measured N deposition at a few sites with spatial interpolation methods, emission inventories, statistical dispersion models, and inferential deposition models. On farmland, the amount of atmospheric N deposition is low relative to the amount of N applied as manure or fertilizer. Therefore, we used the sum of N deposition and N application for farmland species. For simplicity, we speak of N deposition throughout the paper for farmland species, too. Data on N application came from Agroscope (Hutchings et al., 2023) for the year 2020. Thus, these values are not directly comparable with values of N deposition for all other species (i.e., for nonfarmland species). However, this should not be problematic because we were mainly interested in the general pattern of the relationship between N deposition and bird density. We dropped survey areas with missing N data and were left with 2169 areas for analyses.

### Guild groups

We focused on guilds of birds rather than single species, so species were grouped by main habitat (habitat), nesting site (nesting site), migration distance (migration), and main food during breeding season (food) according to the classification of the Swiss Ornithological Institute (Strebel et al., 2020) (Table 1; Appendix S1). To reduce the number of guilds and to examine our assumption, we combined cavity, niche, and open nesting in trees and high bushes in the guild higher‐site‐nesting species. Birds nesting on or near the ground (including the common kingfisher [*Alcedo atthis*], which build burrows near the ground) were grouped in the guild ground‐nesting species. The cuckoo (*Cuculus canorus*) did not fit into these guilds and was ignored relative to the nesting site variable. Short‐distance migrant and resident birds were combined because it is often difficult to assign a species to one of these 2 groups.

**TABLE 1:** Ecological guilds used to group species and their estimated relationships between territory number and N deposition (*n* = 112 species).

Nesting site (number of species)	Migration (number of species)	Food (number of species)^a^	Habitat (number of species)
Ground nesting (37)	Long distance (26)	Insectivorous (invertebrates) (67)	Forest (51)
Higher‐site nesting (74)	Short distance or resident (86)	Herbivorous (plant based) (24)	Farmland (19)
		Vertebrate feeding (10)	Settlement (8)
		Omnivores (mixed diet) (11)	Wetland (12)
			Alpine habitat (9)
			Several habitats^b^ (13)

^a^ Insectivorous, diet of invertebrates, for example, insects, spiders, snails, and worms; herbivorous, diet of plant parts, for example, seeds or leaves of water plants; vertebrate, diet of, for example, fish, rodents, amphibians, and small reptiles; omnivorous, generalist diet of various food types. For species guild assignments, see Appendix S1.

^b^ Species that could not be attributed to a single habitat type.

### Model analyses

All analyses were run in R 4.2.2 (R Development Core Team, 2021). Territory number per survey area was the dependent variable and was analyzed with a zero‐inflated negative binomial model. We used Bayesian statistics and the function brm from the package brms (Bürkner, 2018) with default weakly informative priors.

We first tried to fit a multispecies model that included guilds as covariates and species as random factor. But this model did not converge, so we ran a separate model per species and graphically summed the results per guild. These models included all habitat variables as covariates (centered and scaled to 1 SD) (Appendix S2) and year as a random factor. The variables rocks, wetlands, and shoreline contained a lot of zeros; thus, we changed these to presence or absence. To improve model fit, we removed the variable grassland (habitat variables mostly added to one [i.e., one of them was redundant]) and used the logarithm of N; quadratic effects of elevation, forest, arable land, and roads; and 3 polynomials of log N. For bird species mainly living in alpine habitats (territories above 1000 m asl), we excluded the variable arable land because there is almost no arable land at high elevations. Markov chain Monte Carlo settings were adjusted where needed (increasing number of iterations and reducing step size).

Model fit was assessed using posterior predictive model checking and residual plots, including checking for spatial autocorrelation (semivariograms and bubble plots), which indicated no strong autocorrelation (Korner‐Nievergelt et al., 2015).

For effect plots, covariates were set to their species‐specific mean and factors were set to the most frequent level. Uncertainty intervals were calculated using the 2.5% and 97.5% quantiles of the marginal posterior distributions.

### Calculating linear relationships

Because we used third order polynomials of N, the effect size of N was not easily comparable among species. Thus, per species, we first drew the (third order polynomial) regression line of the number of breeding pairs versus N deposition for 6 ranges of elevation (Appendix S3). Lines of elevation ranges with fewer than 5 data points were omitted. The regression lines were clipped to observed N values per elevation range. From these regression lines, we used the straight line through the fitted values at the 15 and 85 percentiles of the N values (per elevation range, see small dots on regression lines in Appendix S3). The slope was expressed as the change in breeding pairs per change in 10 kg N/ha/year (40 kg N/ha/year for farmland species because the maximal N deposition is 4 times higher in farmland birds than all other species). Second, the slope values were averaged across elevation ranges (proportion of territories used as weights) to yield an (absolute) linear relationship. Third, to make these linear relationships comparable among species, the slope of the (absolute) linear relationship was divided by the mean territory number to yield a relative relationship between territory number and N. The uncertainty from the model was propagated in the absolute and the relative relationship because we performed the averaging for each draw from the joint posterior distribution.

Absolute and relative relationships were only calculated for species with a (mostly) monotonously increasing or decreasing relationship between territory number and N deposition. For species with a clear minima or maxima at intermediate N values, no meaningful linear relationship could be calculated.

## RESULTS

For 61 of 112 studied species, the relationship between territory number and N deposition was monotonously increasing or decreasing. The effect curves of the other 51 species showed at least one minimum or maximum. For 15 of these species, the minima or maxima were at low or high N values such that the regression line was mostly increasing or decreasing. Therefore, we calculated a linear relationship for them as well. For the remaining 36 species, no linear relationship could be stated.

Of the 76 species displaying a linear relationship, 55 showed a negative and 21 a positive relationship. Looking at well‐supported linear relationships only (0 outside the 95% uncertainty interval), 32 showed a negative and 13 a positive correlation of N with breeding numbers. For 31 species, the slope of the relationship was less well supported.

**FIGURE 1:**
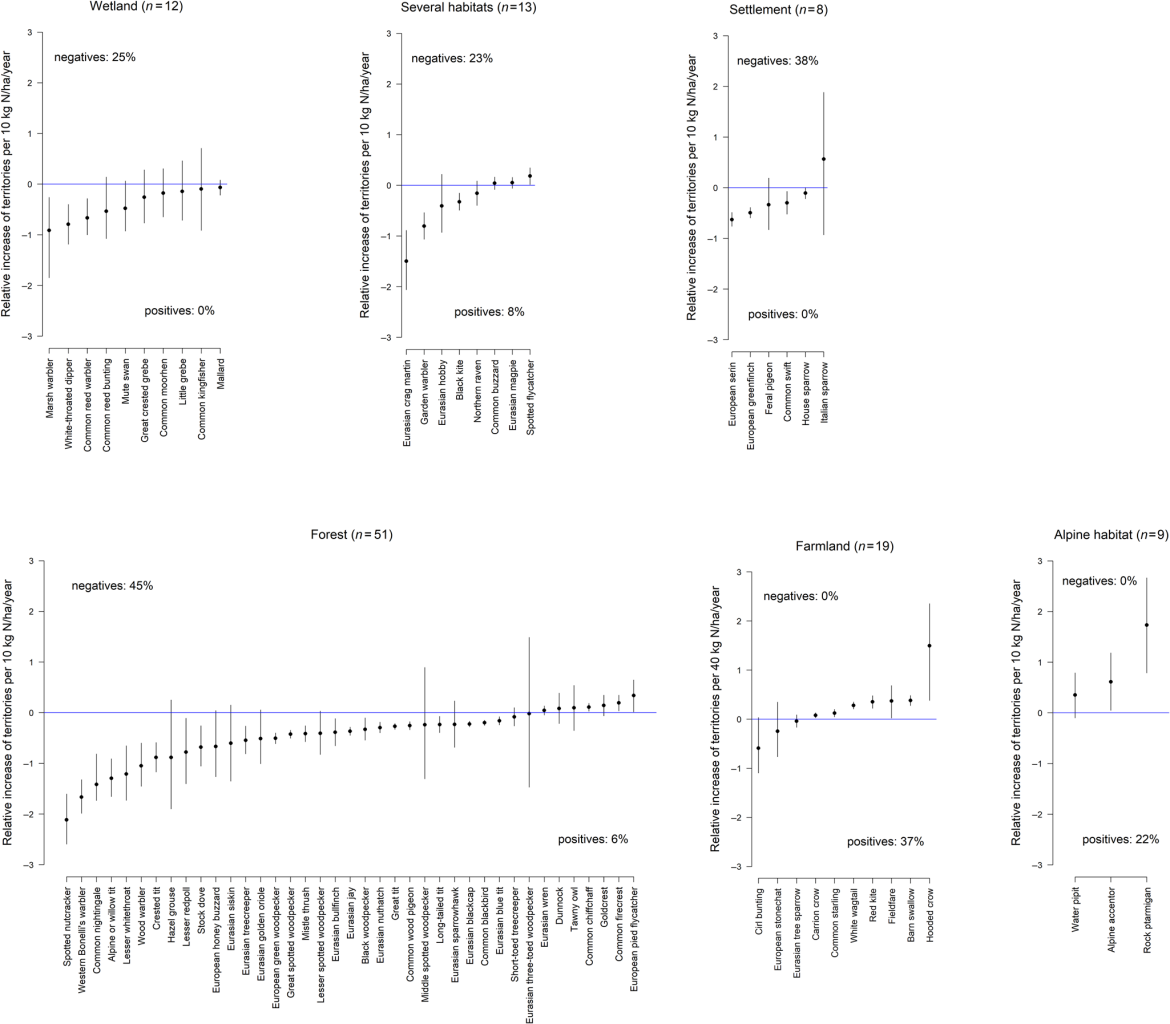
Relative linear relationship of N deposition to territory density for species in 6 habitat guilds. Negative and positive percentages summarize the number of well‐supported (95% uncertainty interval does not include zero) negative and positive relationships as a proportion of all species (with and without linear relationships) in the guild. Percentage of species not shown because they showed no linear relationship: forest, 12 out of 51 (24%); settlement, 2 out of 8 (25%); alpine, 6 out of 9 (67%); several habitats, 5 out of 13 (38%); farmland, 9 out of 19 (47%); wetland, 2 out of 12 (17%).

Inspecting the association of N deposition with territory numbers for different habitat guilds revealed that forest, wetland, and human settlement species and species that could not be attributed to a single guild (several habitats) showed more often a negative than a positive association (Figure 1). For alpine species, most relationships were positive. For farmland birds, relationships were positive or had a maximum around 40 kg N/year/ha.

**FIGURE 2:**
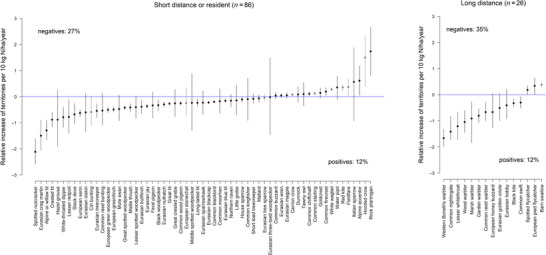
Relative linear relationship of N deposition to territory density for birds in 2 migration guilds (gray lines, farmland birds [relative increase of territories is per 40 kg N/ha/year; see “METHODS”]). Negative and positive percentages summarize the number of well‐supported (95% uncertainty interval does not include zero) negative and positive relationships as a proportion of all species (with and without linear relationships) in the guild. Percentage of species not shown because they showed no linear relationship per guild: short‐distance migration or resident, 25 out of 86 (29%); long‐distance migration, 11 out of 26 (42%).

**FIGURE 3:**
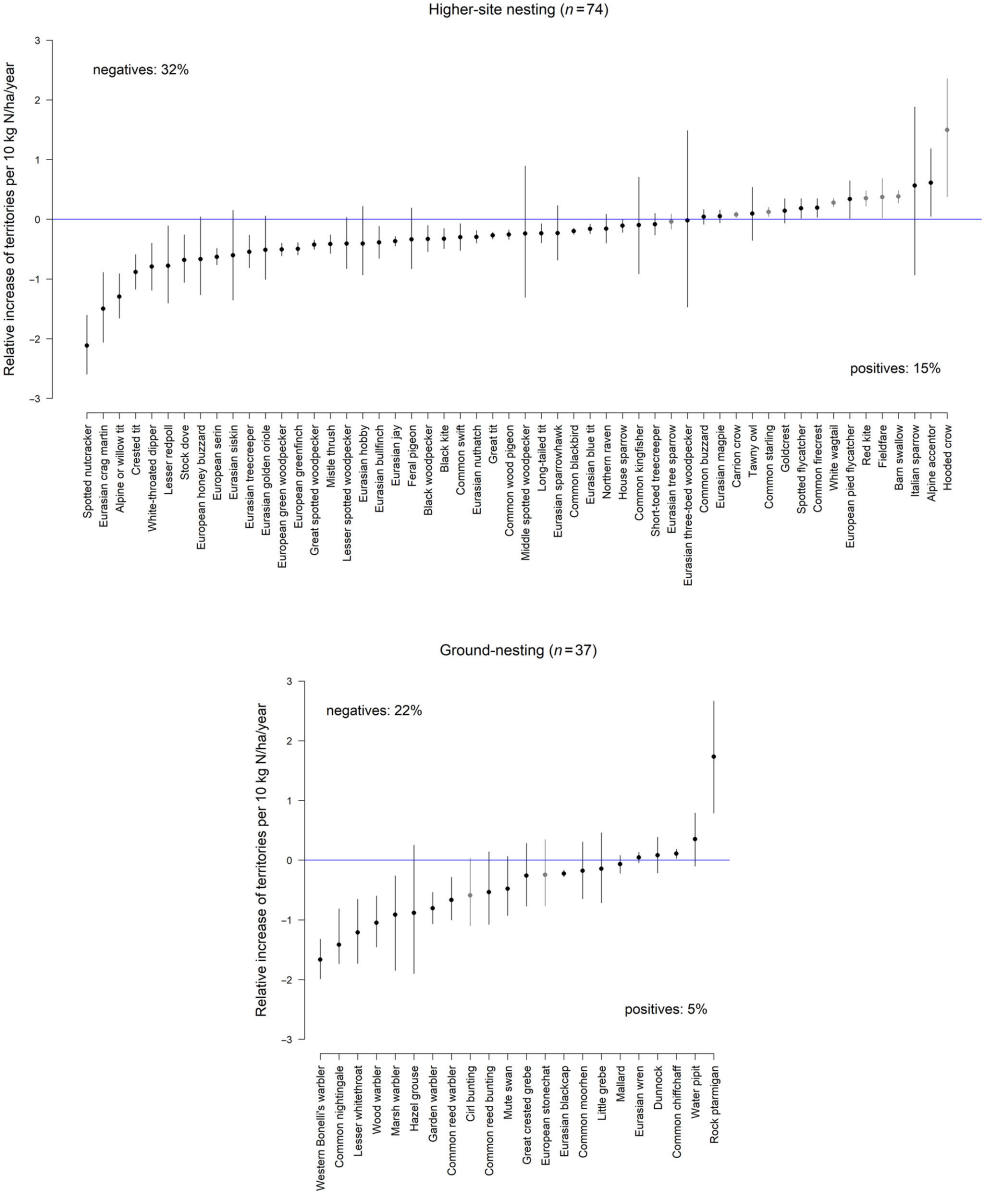
Relative linear relationship of N deposition to territory density for ground‐nesting and higher‐site nesting birds (gray, farmland birds [relative increase of territories is per 40 kg N/ha/year, see “METHODS”]). Negative and positive percentages summarize the number of well‐supported (95% uncertainty interval does not include zero) negative and positive relationships as proportion of all species (with and without linear relationships) in the guild. Percentage of species not shown because they showed no linear relationship per guild: higher‐site nesting, 20 of 74 species (27%); ground nesting, 15 of 37 species (41%).

**FIGURE 4:**
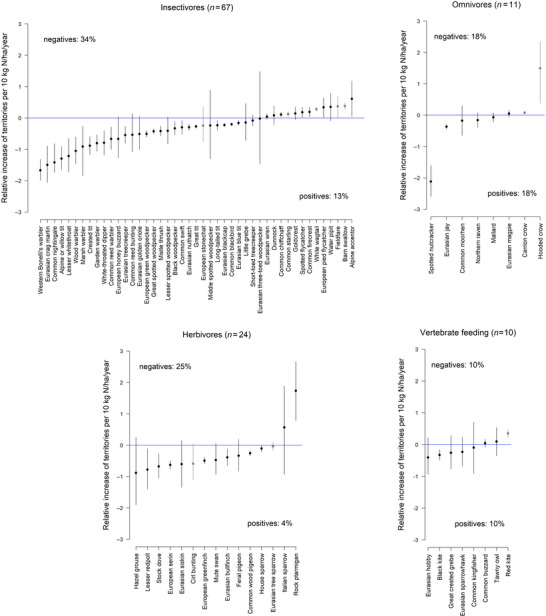
Relative linear relationship of N deposition to territory density for birds in 4 food guilds (gray, farmland birds relative increase of territories is per 40 kg N/ha/year, see “METHODS”). Negative and positive percentages summarize the number of well‐supported (95% uncertainty interval does not include zero) negative and positive relationships as a proportion of all species (with and without linear relationships) in the guild. Percentage of species not shown because they showed no linear relationship per guild: insectivores, 22 out of 67 (33%); vertebrate feeding, 2 out of 10 (20%); herbivores, 9 out of 24 (38%); omnivores, 3 out of 11 (27%).

Long‐distance migratory birds showed slightly more negative correlations between N deposition and territory numbers than short‐distance migrant or resident birds (Figure 2). We found a higher proportion of negative correlations between N deposition and territory numbers in ground‐nesting bird species than in higher‐site‐nesting species (Figure 3). Finally, insectivores and herbivores showed a higher proportion of species with negative correlation between territory density and N deposition than the guilds of omnivores or vertebrate‐feeding species (Figure 4).

## DISCUSSION

Overall, we found a negative correlation of bird territory numbers with N deposition in Switzerland. High N deposition has a fundamental effect on different habitats through its effects on soil chemistry and vegetation structure such that a plethora of cascading effects of N deposition on any species may ensue. Here, we presented correlative observations between N deposition and breeding bird numbers. We are aware that N deposition and its cascading effects will not always be the causal reason for (correlated) bird territory numbers. Other confounding variables may correlate with territory numbers and N deposition—the latter especially because N deposition was itself modeled. For example, the presence of farms was included in the N deposition model (Rihm & Achermann, 2016) and led to a positive association between, for example, barn swallows (*Hirundo rustica*) and N deposition because of the presence of farms (where barn swallows breed) (see discussion on farmland birds below). However, the observed correlations showed valuable overall patterns regarding potential effects of N deposition (and its cascading effects). Therefore, we presented these correlations and speculated about possible general causes for the observed patterns, but detailed studies for each species would be needed to determine the direct and indirect mechanisms linking N deposition to bird abundance.

Territory numbers of two thirds (55) of the 76 species that showed a (mostly) monotonous relationship were negatively correlated with N deposition. Considering only statistically robust linear relationships, 32 of 45 species were negatively correlated with N, and only 13 were positively correlated. This result is in line with other studies demonstrating negative consequences of N on biodiversity. Different explanations for the negative correlations of N input and bird numbers are conceivable, from altered habitat structure influencing nesting sites to accessibility and availability of food (invertebrates as well as plant food). Nitrogen is an important nutrient for plants. It enhances growth and density of vegetation, but it also reduces plant diversity and leads to a homogenization of vegetation (Wright et al., 2018), especially in grasslands (Bobbink et al., 2010). It is obvious that this could directly affect nesting attempts (e.g., of skylarks [Jenny, 1990; Wilson et al., 1997]) and food sources for herbivorous bird species. Further, N input can impair diversity and abundance of butterflies (Roth et al., 2021) and other insects (Nijssen et al., 2001; Vogels et al., 2017) and, thus, probably results in poorer food availability for insectivorous bird species.

Our results showed that insectivorous bird numbers were more negatively associated with high N deposition than birds foraging on vertebrates and omnivorous birds, suggesting a lager negative effect of N on insectivorous species. The herbivorous bird guild also contained many species with a negative linear relationship of territory density with increasing N deposition. Species that are food generalists and feed on carrion or anthropogenic food sources, for example, have an advantage in the modern landscape with their intensive farming and growing settlements and streets. Birds that rely on insects and other invertebrates or on plant‐based food, such as seeds or grains, have overall more problems with high N deposition. As mentioned above, high N inputs can negatively affect insects, which are food for many bird species. Nitrogen input also leads to the loss of specialized plant species, reducing floral diversity and thus, especially outside the main flowering period, impairing food supply. Further, dense vegetation can hinder accessibility of invertebrates for hunting birds (Martinez et al., 2010; Schaub et al., 2010).

Most bird species belonging to the habitat guilds forest, wetlands, and settlements and species that could not be attributed to a single guild (several habitats) showed a negative correlation with N deposition, but birds living in alpine habitats or on farmland did not.

High inputs of N favor nitrophilous plant species. In forests, N deposition therefore changes vegetation structure, favoring thickets of brambles or nettles (Flückiger & Braun, 2004) and reducing available nesting and foraging habitats for birds specialized on sparsely vegetated ground, such as the wood warbler (Pasinelli et al., 2016; Scheibler, 2015). In contrast, this could be an advantage for birds preferring dense undergrowth to hide and breed in, such as Eurasian wren (*Troglodytes troglodytes*) and dunnock (*Prunella modularis*), both of which showed a (weak) positive response to higher N deposition. Birds may also be negatively affected by N deposition in that it could cause food shortages. High N deposition reduces, for example, the availability for birds of earthworms in the upper soil layer (Onrust et al., 2019). In wetlands, diversity and compositions of water organisms (from small organisms to fish) are affected by high N levels (Camargo & Alonso, 2006), which may cause food shortage for water birds.

Alpine species showed a positive relationship with N. However, a trend could only be calculated for 3 out of 8 species. Inferences should therefore be made with care. Alpine habitats are characterized by low levels of N and thus low primary production. Higher N deposition possibly leads to increased vegetation growth, providing more food and habitat for invertebrates. Higher densities of invertebrates mean more food for alpine bird species and their nestlings. However, changes in habitat structure have many different effects on birds (and the entire ecosystem), for example, in terms of shelter, breeding habitat, and food sources. From a conservation point of view, it is obviously not desirable to artificially increase the naturally low primary production in alpine habitats.

For farmland birds, territory numbers of 7 species were positively correlated with N input and 3 species showed a negative correlation (although with quite large uncertainty). Eight of the 9 farmland species without a linear relationship (common linnet [*Linaria cannabina*], common kestrel [*Falco tinnunculus*], common whitethroat [*Curruca communis*], Eurasian skylark [*Alauda arvensis*], Eurasian wryneck [*Jynx torquilla*], red backed shrike [*Lanius collurio*], tree pipit [*Anthus trivialis*], yellowhammer [*Emberiza citronella*]) seemed to prefer intermediate amounts of N; territory density declined at higher levels of N input (Appendix S3). Many species dependent on extensively used farmland (i.e., species of the traditional landscapes preceding the agricultural intensification) are expected to show a negative relationship with N deposition. Although most such species are now rare in Switzerland, and therefore excluded from our analyses, cirl bunting (*Emberiza cirlus*) and European stonechat (*Saxicola rubicola*) were analyzed and showed a negative correlation with N (farmland species excluded due to now low population size were quail [*Coturnix coturnix*], corn crake [*Crex crex*], meadow pipit [*Anthus pratensis*], gray partridge [*Perdix perdix*], northern lapwing [*Vanellus vanellus*], western yellow wagtail [*Motacilla flava*], and corn bunting [*Emberiza calandra*]). The barn swallow, hooded crow (*Corvus cornix*), and red kite (*Milvus milvus*)—species that showed a positive correlation with N—hunt their food over large areas, whereas farmland species that showed no or a negative linear relationship with N often have smaller territories. The larger the home range, the higher the probability that good hunting habitat is included. Fertilization of meadows causes faster vegetation growth and therefore more mowing events, which is detrimental to invertebrates as well as meadow‐breeding birds. However, freshly cut, intensively used grassland provides feeding opportunities (insects and rodents, some killed by the cutting, are easily accessible in the short grass) for some species, such as hooded crow, starling (*Sturnus vulgaris*), and red kite. The positive correlation seen in the barn swallow comes from the fact that this species nests in barns and stables and N deposition is high in regions with high livestock densities.

In line with our expectation, we found a difference in the correlation of territory numbers with N deposition between the nesting site guilds. The linear relationships in ground‐nesting species were mainly negative, whereas in higher‐site nesting birds this association was less pronounced (22% and 5% in ground‐nesting birds vs. 32% and 15% in higher‐site‐nesting birds), suggesting a more negative effect of N deposition on ground‐breeding birds than on higher‐site‐nesting birds. High input of N enhances vegetation growth and density and thus alters breeding habitat for ground‐nesting bird species, such as warblers (Pasinelli et al., 2016). In meadows, higher N deposition leads to more frequent mowing, which damages nests and harms birds.

A small difference in the correlation of territory numbers with N deposition was also found among the migration guilds (35% negative and 12% positive relationships in long‐distance migrant species vs. 27% and 12% in resident or short‐distance migrant species). These results met our expectation of a more negative effect of N deposition on long‐distance migrant birds because they contain many insectivorous species, compared with the short‐distance migration or resident guild. High N input can negatively affect insects, and altered species composition or reduced biomass of insect species means degraded food resources for insectivorous birds.

The problem of high N deposition for biodiversity has been known for decades, but the topic has only lately received wider attention in the media (Agroscope, 2023; bz, 2023; Kurt Seiler & Roger Biedermann, 2023; SRF, 2023) and politics (Bundesamt für Landwirtschaft [BLW], 2023; Bundesamt für Umwelt [BAFU], 2024). However, recent initiatives for mandatory implementation of unfertilized areas on arable land in Switzerland and the European Union were canceled (Fabiana Luca, 2024; Schweizer Bauer, 2024). Negative consequences of N deposition on humans, such as poor‐quality drinking water, have received more attention in the past. In Switzerland, projects were started in the early 2000s to reduce high amounts of N in groundwater, albeit with limited success; many areas still show high levels of N in the groundwater. However, there have been some improvements: levels of N in some rivers and lakes have been reduced or stabilized and N deposition has decreased in the last about 20 years in Switzerland (Knapp & Posch, 2022). But N deposition is still above critical loads in most parts of Switzerland. Nitrogen emissions intensify climate change, affecting both humans and biodiversity. Hence, although these direct negative effects on humans should be enough reason to reduce N input in our environment, our results add a reason to limit N emissions by showing for the first time the negative relationships of high N levels with territory numbers of a variety of bird species. Considering biodiversity declines in Switzerland and worldwide, there is an urgent need to significantly increase the efforts to lower N inputs into the environment.

## Supporting information



Supporting Information

Supporting Information

Supporting Information

## References

[cobi70114-bib-0001] Agroscope . (2023, November 28). Herausforderung Nährstoffverluste – Agroscope unterstützt die landwirtschaftliche Praxis [Press release]. Agroscope.

[cobi70114-bib-0002] Almond, R. , Grooten, M. , Juffe Bignoli, D. , & Petersen, T. (2022). Living planet report—Building a nature‐positive society. WWF.

[cobi70114-bib-0003] Billeter, R. , Liira, J. , Bailey, D. , Bugter, R. , Arens, P. , Augenstein, I. , Aviron, S. , Baudry, J. , Bukacek, R. , Burel, F. , Cerny, M. , De Blust, G. , De Cock, R. , Diekötter, T. , Dietz, H. , Dirksen, J. , Dormann, C. , Durka, W. , Frenzel, M. , … Edwards, P. J. (2008). Indicators for biodiversity in agricultural landscapes: A pan‐European study. Journal of Applied Ecology, 45(1), 141–150.

[cobi70114-bib-0004] Bobbink, R. , Hicks, K. , Galloway, J. , Spranger, T. , Alkemade, R. , Ashmore, M. , Bustamante, M. , Cinderby, S. , Davidson, E. , Dentener, F. , Emmett, B. , Erisman, J. W. , Fenn, M. , Gilliam, F. , Nordin, A. , Pardo, L. , & de Vries, W. (2010). Global assessment of nitrogen deposition effects on terrestrial plant diversity: A synthesis. Ecological Applications, 20(1), 30–59. 20349829 10.1890/08-1140.1

[cobi70114-bib-0005] Bobbink, R. , Hornung, M. , & Roelofs, J. G. M. (1998). The effects of air‐borne nitrogen pollutants on species diversity in natural and semi‐natural European vegetation. Journal of Ecology, 86(5), 717–738.

[cobi70114-bib-0006] Brondízio, E. S. , Settele, J. , Díaz, S. , & Ngo, H. T. (Eds.). (2019). The global assessment report of the Intergovernmental Science‐Policy Platform on Biodiversity and Ecosystem Services. Intergovernmental Science‐Policy Platform on Biodiversity and Ecosystem Services (IPBES).

[cobi70114-bib-0007] Bundesamt für Landestopografie . (2005). DHM25. Das digitale Höhenmodell der Schweiz. Produktinformation . Swisstopo.

[cobi70114-bib-0008] Bundesamt für Landestopografie . (2007). VECTOR25. Das digitale Landschaftsmodell der Schweiz. Produkteinformation . Swisstopo.

[cobi70114-bib-0009] Bundesamt für Landwirtschaft (BLW) . (2023, December 4). Bundesrat passt nach der Vernehmlassung mehrere Agrarverordnungen an [Press release]. https://www.blw.admin.ch/blw/de/home/services/medienmitteilungen.msg‐id‐98414.html

[cobi70114-bib-0010] Bundesamt für Statistik . (2014). Arealstatistik nach Nomenklatur 2004 – Standard: GEOSTAT‐Datenbeschreibung . Author.

[cobi70114-bib-0011] Bundesamt für Statistik . (Ed.). (2015). Statistik der Schweiz, Fachbereich 2, Raum und Umwelt. Die Bodennutzung in der Schweiz . Auswertungen und Analysen.

[cobi70114-bib-0012] Bundesamt für Umwelt (BAFU) . (2024, May 1). Bundesrat verabschiedet Prüfbericht zur Verbesserung der Nährstoffsituation im Wald [Press release]. https://www.admin.ch/gov/de/start/dokumentation/medienmitteilungen.msg‐id‐100873.html

[cobi70114-bib-0013] Bürkner, P.‐C. (2018). Advanced Bayesian multilevel modeling with the R package brms. The R Journal, 10(1), 395–411.

[cobi70114-bib-0014] bz . (2023, July 4). Der Wald in der Region Basel leidet unter der Gülle—jetzt wollen die Waldeigentümer mit den Bauern reden [Press release]. https://www.bzbasel.ch/basel/baselland/landwirtschaft‐der‐wald‐leidet‐unter‐den‐ausscheidungen‐von‐kuehen‐jetzt‐wollen‐die‐waldeigentuemer‐mit‐den‐bauern‐reden‐ld.2484052

[cobi70114-bib-0015] Camargo, J. A. , & Alonso, A. (2006). Ecological and toxicological effects of inorganic nitrogen pollution in aquatic ecosystems: A global assessment. Environment International, 32(6), 831–849. 16781774 10.1016/j.envint.2006.05.002

[cobi70114-bib-0016] Clark, C. M. , & Tilman, D. (2008). Loss of plant species after chronic low‐level nitrogen deposition to prairie grasslands. Nature, 451(7179), 712–715. 18256670 10.1038/nature06503

[cobi70114-bib-0017] De Schrijver, A. , De Frenne, P. , Ampoorter, E. , van Nevel, L. , Demey, A. , Wuyts, K. , & Verheyen, K. (2011). Cumulative nitrogen input drives species loss in terrestrial ecosystems. Global Ecology and Biogeography, 20(6), 803–816.

[cobi70114-bib-0018] Ewing, S. R. , Baxter, A. , Wilson, J. D. , Hayhow, D. B. , Gordon, J. , Des Thompson, B. A. , Whitfield, D. P. , & van der Wal, R. (2020). Clinging on to alpine life: Investigating factors driving the uphill range contraction and population decline of a mountain breeding bird. Global Change Biology, 26(7), 3771–3787. 32350939 10.1111/gcb.15064

[cobi70114-bib-0019] Fabiana, L. (2024, February 13). Brussels adopts waiver on uncultivated CAP land through 2024. *eunews*.

[cobi70114-bib-0020] Flückiger, W. , & Braun, S. (2004). Wie geht es unserem Wald? Ergebnisse aus Dauerbeobachtungsflächen von 1984 bis 2004. Bericht 2 . Institut für Angewandte Pflanzenbiologie.

[cobi70114-bib-0021] Gilliam, F. S. (2006). Response of the herbaceous layer of forest ecosystems to excess nitrogen deposition. Journal of Ecology, 94(6), 1176–1191.

[cobi70114-bib-0022] Hautier, Y. , Niklaus, P. A. , & Hector, A. (2009). Competition for light causes plant biodiversity loss after eutrophication. Science, 324(5927), 636–638. 19407202 10.1126/science.1169640

[cobi70114-bib-0023] Hohl, S. , Hochreutener, M. , Lüthy, L. , Roth, M. , & Spaar, R. (2021). Artenförderung Kiebitz in der Wauwiler Ebene. Kanton Luzern—Jahresbericht 2021 . Schweizerische Vogelwarte.

[cobi70114-bib-0024] Humbert, J.‐Y. , Dwyer, J. M. , Andrey, A. , & Arlettaz, R. (2015). Impacts of nitrogen addition on plant biodiversity in mountain grasslands depend on dose, application duration and climate: A systematic review. Global Change Biology, 22(1), 110–120. 26010833 10.1111/gcb.12986

[cobi70114-bib-0025] Hutchings, C. , Spiess, E. , & Prasuhn, V. (2023). Abschätzung diffuser Stickstoff‐ und Phosphoreinträge in die Gewässer der Schweiz mit MODIFFUS 3.1,Stand 2020. Agroscope Science, 10.34776/as155g

[cobi70114-bib-0026] Jenny, M. (1990). Territorialität und Brutbiologie der Feldlerche Alauda arvensis in einer intensiv genutzten Agrarlandschaft. Journal für Ornithologie, 131(3), 241–265.

[cobi70114-bib-0027] Kleijn, D. , Kohler, F. , Báldi, A. , Batáry, P. , Concepción, E. D. , Clough, Y. , Díaz, M. , Gabriel, D. , Holzschuh, A. , Knop, E. , Kovács, A. , Marshall, E. J. P. , Tscharntke, T. , & Verhulst, J. (2009). On the relationship between farmland biodiversity and land‐use intensity in Europe. Proceedings of the Royal Society B: Biological Sciences, 276(1658), 903–909. 10.1098/rspb.2008.1509 PMC2664376 19019785

[cobi70114-bib-0028] Knapp, D. , & Posch, T. (2022). Veränderung der Stickstoff‐ zu Phosphor‐Verhältnisse in Seen—Mögliche Konsequenzen für die Struktur von Nahrungsnetzen in Schweizer Seen: Projekt im Auftrag des Bundesamtes für Umwelt BAFU, Bern . Limnologische Station, Institut für Pflanzen‐ und Mikrobiologie, Universität Zürich.

[cobi70114-bib-0029] Knaus, P. , Antoniazza, S. , Wechsler, S. , Guélat, J. , Kéry, M. , Strebel, N. , & Sattler, T. (Eds.). (2018). Schweizer Brutvogelatlas 2013–2016: Verbreitung und Bestandsentwicklung der Vögel in der Schweiz und im Fürstentum Liechtenstein. Schweizerische Vogelwarte.

[cobi70114-bib-0030] Koordinationsstelle BDM . (2014). Biodiversitätsmonitoring Schweiz BDM: Beschreibung der Methoden und Indikatoren (Umwelt‐Wissen No. 1410). www.bafu.admin.ch/uw‐uw‐1410‐d

[cobi70114-bib-0031] Korner‐Nievergelt, F. , Roth, T. , Felten, S. v. , Guélat, J. , Almasi, B. , & Korner‐Nievergelt, P. (2015). Bayesian data analysis in ecology using linear models with R, BUGS, and Stan. Academic Press.

[cobi70114-bib-0032] Liu, X. , Shi, X. , & Zhang, S. (2021). Soil abiotic properties and plant functional diversity co‐regulate the impacts of nitrogen addition on ecosystem multifunctionality in an alpine meadow. The Science of the Total Environment, 780, Article 146476. 33773353 10.1016/j.scitotenv.2021.146476

[cobi70114-bib-0033] Martinez, N. , Jenni, L. , Wyss, E. , & Zbinden, N. (2010). Habitat structure versus food abundance: The importance of sparse vegetation for the common redstart *Phoenicurus phoenicurus* . Journal of Ornithology, 151(2), 297–307.

[cobi70114-bib-0034] Maskell, L. C. , Smart, S. M. , Bullock, J. M. , Thompson, K. E. , & Stevens, C. J. (2010). Nitrogen deposition causes widespread loss of species richness in British habitats. Global Change Biology, 16(2), 671–679.

[cobi70114-bib-0035] Nijssen, M. , Alders, K. , van der Smissen, N. , & Esselink, H. (2001). Effects of grass‐encroachment and grazing management on carabid assemblages of dry dune grasslands. Proceedings of the Section Experimental and Applied Entomology of the Netherlands Entomological Society, 12, 113–120.

[cobi70114-bib-0036] Onrust, J. , Wymenga, E. , Piersma, T. , & Olff, H. (2019). Earthworm activity and availability for meadow birds is restricted in intensively managed grasslands. Journal of Applied Ecology, 56, 1333–1342.

[cobi70114-bib-0037] Pasinelli, G. , Grendelmeier, A. , Gerber, M. , & Arlettaz, R. (2016). Rodent‐avoidance, topography and forest structure shape territory selection of a forest bird. BMC Ecology, 16, Article 24. 10.1186/s12898-016-0078-8 27160928 PMC4860761

[cobi70114-bib-0038] Pöyry, J. , Carvalheiro, L. G. , Heikkinen, R. K. , Kühn, I. , Kuussaari, M. , Schweiger, O. , Valtonen, A. , van Bodegom, P. M. , & Franzén, M. (2017). The effects of soil eutrophication propagate to higher trophic levels. Global Ecology and Biogeography, 26(1), 18–30.

[cobi70114-bib-0039] R Development Core Team . (2021). R: A language and environment for statistical computing. R Foundation for Statistical Computing.

[cobi70114-bib-0040] Rihm, B. , & Achermann, B. (2016). Critical loads of nitrogen and their exceedances. Swiss contribution to the effects‐oriented work under the Convention on Long‐range Transboundary Air Pollution (UNECE) (Environmental Studies no. 1642.). Federal Office for the Environment (FOEN). 10.13140/RG.2.2.16281.01124

[cobi70114-bib-0041] Roth, T. , Kohli, L. , Rihm, B. , & Achermann, B. (2013). Nitrogen deposition is negatively related to species richness and species composition of vascular plants and bryophytes in Swiss mountain grassland. Agriculture, Ecosystems and Environment, 178, 121–126.

[cobi70114-bib-0042] Roth, T. , Kohli, L. , Rihm, B. , Meier, R. , & Amrhein, V. (2021). Negative effects of nitrogen deposition on Swiss butterflies. Conservation Biology: The Journal of the Society for Conservation Biology, 35(6), 1766–1776. 33829544 10.1111/cobi.13744

[cobi70114-bib-0043] Schaub, M. , Martinez, N. , Tagmann‐Ioset, A. , Weisshaupt, N. , Maurer, M. L. , Reichlin, T. S. , Abadi, F. , Zbinden, N. , Jenni, L. , & Arlettaz, R. (2010). Patches of bare ground as a staple commodity for declining ground‐foraging insectivorous farmland birds. PLoS ONE, 5(10), Article e13115. 20949083 10.1371/journal.pone.0013115 PMC2950849

[cobi70114-bib-0044] Scheibler, D. (2015). Nitrogen deposition and forest clearings in wood warbler habitats in Switzerland (MS thesis). Universität Zürich.

[cobi70114-bib-0045] Schweizer Bauer . (2024). 3,5% BFF auf Ackerland kommen nicht . Author.

[cobi70114-bib-0046] Schweizer Radio und Fernsehen (SRF) . (2023, December 14). Schädliche Gülle—Amtlich tolerierte Umweltverschmutzung . Schweizer Radio und Fernsehen.

[cobi70114-bib-0047] Seiler, K. , & Biedermann, R. (2023). Stickstoff: Wege aus der Sackgasse. Aqua Viva, 2023(4), 30–36.

[cobi70114-bib-0048] Soons, M. B. , Hefting, M. M. , Dorland, E. , Lamers, L. P. , Versteeg, C. , & Bobbink, R. (2017). Nitrogen effects on plant species richness in herbaceous communities are more widespread and stronger than those of phosphorus. Biological Conservation, 212, 390–397.

[cobi70114-bib-0049] Stevens, C. J. , David, T. I. , & Storkey, J. (2018). Atmospheric nitrogen deposition in terrestrial ecosystems: Its impact on plant communities and consequences across trophic levels. Functional Ecology, 32(7), 1757–1769.

[cobi70114-bib-0050] Stevens, C. J. , Dise, N. B. , Gowing, D. J. G. , & Mountford, J. O. (2006). Loss of forb diversity in relation to nitrogen deposition in the UK: Regional trends and potential controls. Global Change Biology, 12(10), 1823–1833.

[cobi70114-bib-0051] Stevens, C. J. , Dise, N. B. , Mountford, J. O. , & Gowing, D. J. (2004). Impact of nitrogen deposition on the species richness of grasslands. Science, 303(5665), 1876–1879. 15031507 10.1126/science.1094678

[cobi70114-bib-0052] Stevens, C. J. , Lind, E. M. , Hautier, Y. , Harpole, W. S. , Borer, E. T. , Hobbie, S. , Seabloom, E. W. , Ladwig, L. , Bakker, J. D. , Chu, C. , Collins, S. , Davies, K. F. , Firn, J. , Hillebrand, H. , La Pierre, K. J. , MacDougall, A. , Melbourne, B. , McCulley, R. L. , Morgan, J. , … Wragg, P. D. (2015). Anthropogenic nitrogen deposition predicts local grassland primary production worldwide. Ecology, 96(6), 1459–1465.

[cobi70114-bib-0053] Strebel, N. , Schmid, H. , Wechsler, S. , & Sattler, T. (2020). Update der Gildeneinteilung der Schweizer Brutvögel . Interner Bericht.

[cobi70114-bib-0054] Suding, K. N. , Collins, S. L. , Gough, L. , Clark, C. , Cleland, E. E. , Gross, K. L. , Milchunas, D. G. , & Pennings, S. (2005). Functional‐ and abundance‐based mechanisms explain diversity loss due to N fertilization. Proceedings of the National Academy of Sciences of the United States of America, 102(12), 4387–4392. 15755810 10.1073/pnas.0408648102 PMC555488

[cobi70114-bib-0055] Tilman, D. (1993). Species richness of experimental productivity gradients: How important is colonization limitation? Ecology, 74(8), 2179–2191.

[cobi70114-bib-0056] Tresch, S. , Roth, T. , Schindler, C. , Hopf, S.‐E. , Remund, J. , & Braun, S. (2023). The cumulative impacts of droughts and N deposition on Norway spruce (*Picea abies*) in Switzerland based on 37 years of forest monitoring. The Science of the Total Environment, 892, Article 164223. 37236453 10.1016/j.scitotenv.2023.164223

[cobi70114-bib-0057] Treseder, K. K. (2008). Nitrogen additions and microbial biomass: A meta‐analysis of ecosystem studies. Ecology Letters, 11(10), 1111–1120. 18673384 10.1111/j.1461-0248.2008.01230.x

[cobi70114-bib-0058] van den Burg, A. (2021). The quality of protein sources for egg production in Tawny Owls (*Strix aluco*) and Eurasian Sparrowhawks (*Accipiter nisus*) [A qualidade das fontes de proteína para a produção de ovos em coruja‐do‐mato (*Strix aluco*) e gavião (*Accipiter nisus*)]. Airo, 29, 467–476.

[cobi70114-bib-0059] Vitousek, P. M. , Aber, J. D. , Howarth, R. W. , Likens, G. E. , Matson, P. A. , Schindler, D. W. , Schlesinger, W. H. , & Tilman, D. G. (1997). Human alteration of the global nitrogen cycle: Sources and consequences. Ecological Applications, 7(3), 737–750.

[cobi70114-bib-0060] Vogels, J. J. , Verberk, W. , Lamers, L. , & Siepel, H. (2017). Can changes in soil biochemistry and plant stoichiometry explain loss of animal diversity of heathlands? Biological Conservation, 212, 432–447.

[cobi70114-bib-0061] Wang, C. , Liu, D. , & Bai, E. (2018). Decreasing soil microbial diversity is associated with decreasing microbial biomass under nitrogen addition. Soil Biology and Biochemistry, 120, 126–133.

[cobi70114-bib-0062] Wilson, J. D. , Evans, J. , Browne, S. J. , & King, J. R. (1997). Territory distribution and breeding success of skylarks *Alauda arvensis* on organic and intensive farmland in southern England. Journal of Applied Ecology, 34, 1462–1478.

[cobi70114-bib-0063] Wright, L. P. , Zhang, L. , Cheng, I. , Aherne, J. , & Wentworth, G. R. (2018). Impacts and effects indicators of atmospheric deposition of major pollutants to various ecosystems—A review. Aerosol and Air Quality Research, 18(8), 1953–1992.

