# Supplementary material for: Effects of nitrogen deposition on territory numbers of breeding birds
Source: Conserv Biol. 2025 Aug 15;39(6):e70114. doi: 10.1111/cobi.70114 (PMC12658931; doi:10.1111/cobi.70114)
Supplement: Supplementary file 1 — Supporting Information [file COBI-39-e70114-s002.docx]

**S1.** 112 studied bird species and their classification into guilds as well as the relative linear relationship plus uncertainty interval of territory number with N deposition of the 76 species for which a linear relationship could be calculated.

| Species name English | species name Latin | Habitat ^a^ | Migration ^b^ | Food ^c^ | nest site ^d^ | relative linear relationship | uncertainty interval | |
| --- | --- | --- | --- | --- | --- | --- | --- | --- |
| Spotted nutcracker | Nucifraga caryocatactes | Wo | R | O | OH | -2.12 | -2.59 | -1.61 |
| Western bonelli's warbler | Phylloscopus bonelli | Wo | L | I | G | -1.67 | -1.99 | -1.32 |
| Eurasian crag martin | Ptyonoprogne rupestris | Sev | S | I | N | -1.5 | -2.06 | -0.89 |
| Common nightingale | Luscinia megarhynchos | Wo | L | I | G | -1.42 | -1.73 | -0.81 |
| Willow tit | Poecile montanus | Wo | R | I | C | -1.29 | -1.66 | -0.91 |
| Lesser whitethroat | Curruca curruca | Wo | L | I | OL | -1.21 | -1.73 | -0.65 |
| Wood warbler | Phylloscopus sibilatrix | Wo | L | I | G | -1.05 | -1.45 | -0.6 |
| Marsh warbler | Acrocephalus palustris | We | L | I | OL | -0.91 | -1.85 | -0.26 |
| Crested tit | Lophophanes cristatus | Wo | R | I | C | -0.88 | -1.17 | -0.59 |
| Hazel grouse | Tetrastes bonasia | Wo | R | P | G | -0.88 | -1.9 | 0.25 |
| Garden warbler | Sylvia borin | Sev | L | I | OL | -0.8 | -1.06 | -0.54 |
| White-throated dipper | Cinclus cinclus | We | R | I | N | -0.79 | -1.19 | -0.4 |
| Lesser redpoll | Acanthis cabaret | Wo | S | P | OH | -0.78 | -1.4 | -0.11 |
| Stock dove | Columba oenas | Wo | S | P | C | -0.68 | -1.05 | -0.26 |
| Common reed warbler | Acrocephalus scirpaceus | We | L | I | OL | -0.67 | -1 | -0.28 |
| European honey buzzard | Pernis apivorus | Wo | L | I | OH | -0.67 | -1.26 | 0.04 |
| European serin | Serinus serinus | S | S | P | OH | -0.63 | -0.76 | -0.49 |
| Eurasian siskin | Spinus spinus | Wo | S | P | OH | -0.6 | -1.35 | 0.15 |
| Cirl bunting | Emberiza cirlus | F | R | P | OL | -0.59 | -1.1 | 0.03 |
| Eurasian treecreeper | Certhia familiaris | Wo | R | I | C | -0.55 | -0.81 | -0.26 |
| Common reed bunting | Emberiza schoeniclus | We | S | I | OL | -0.54 | -1.07 | 0.14 |
| Eurasian golden oriole | Oriolus oriolus | Wo | L | I | OH | -0.51 | -1.01 | 0.06 |
| European green woodpecker | Picus viridis | Wo | R | I | C | -0.51 | -0.61 | -0.4 |
| European greenfinch | Chloris chloris | S | R | P | OH | -0.49 | -0.6 | -0.39 |
| Mute swan | Cygnus olor | We | R | P | G | -0.48 | -0.92 | 0.06 |
| Great spotted woodpecker | Dendrocopos major | Wo | R | I | C | -0.42 | -0.5 | -0.34 |
| Mistle thrush | Turdus viscivorus | Wo | S | I | OH | -0.42 | -0.57 | -0.26 |
| Lesser spotted woodpecker | Dryobates minor | Wo | R | I | C | -0.41 | -0.82 | 0.03 |
| Eurasian hobby | Falco subbuteo | Sev | L | V | OH | -0.41 | -0.93 | 0.22 |
| Eurasian bullfinch | Pyrrhula pyrrhula | Wo | S | P | OH | -0.39 | -0.65 | -0.12 |
| Eurasian jay | Garrulus glandarius | Wo | S | O | OH | -0.37 | -0.45 | -0.28 |
| Feral pigeon | Columba livia domestica | S | R | P | N | -0.34 | -0.83 | 0.19 |
| Black woodpecker | Dryocopus martius | Wo | R | I | C | -0.33 | -0.54 | -0.1 |
| Black kite | Milvus migrans | Sev | L | V | OH | -0.33 | -0.49 | -0.16 |
| Common swift | Apus apus | S | L | I | C | -0.3 | -0.52 | -0.07 |
| Eurasian nuthatch | Sitta europaea | Wo | R | I | C | -0.29 | -0.4 | -0.19 |
| Great tit | Parus major | Wo | S | I | C | -0.27 | -0.33 | -0.21 |
| Great crested grebe | Podiceps cristatus | We | S | V | G | -0.26 | -0.77 | 0.28 |
| Common wood pigeon | Columba palumbus | Wo | S | P | OH | -0.26 | -0.34 | -0.18 |
| European stonechat | Saxicola rubicola | F | S | I | G | -0.25 | -0.76 | 0.34 |
| Middle spotted woodpecker | Dendrocoptes medius | Wo | R | I | C | -0.24 | -1.3 | 0.89 |
| Long-tailed tit | Aegithalos caudatus | Wo | S | I | OH | -0.23 | -0.39 | -0.07 |
| Eurasian sparrowhawk | Accipiter nisus | Wo | S | V | OH | -0.23 | -0.68 | 0.23 |
| Eurasian blackcap | Sylvia atricapilla | Wo | S | I | OL | -0.22 | -0.28 | -0.17 |
| Common blackbird | Turdus merula | Wo | S | I | OH | -0.2 | -0.25 | -0.15 |
| Common moorhen | Gallinula chloropus | We | R | O | OL | -0.18 | -0.65 | 0.31 |
| Eurasian blue tit | Cyanistes caeruleus | Wo | S | I | C | -0.16 | -0.24 | -0.08 |
| Northern raven | Corvus corax | Sev | R | O | N | -0.16 | -0.4 | 0.09 |
| Little grebe | Tachybaptus ruficollis | We | S | I | G | -0.14 | -0.71 | 0.46 |
| House sparrow | Passer domesticus | S | R | P | C | -0.11 | -0.22 | 0 |
| Common kingfisher | Alcedo atthis | We | S | V | C | -0.1 | -0.91 | 0.71 |
| Short-toed treecreeper | Certhia brachydactyla | Wo | R | I | C | -0.08 | -0.26 | 0.1 |
| Mallard | Anas platyrhynchos | We | S | O | G | -0.07 | -0.22 | 0.08 |
| Eurasian tree sparrow | Passer montanus | F | S | P | C | -0.04 | -0.16 | 0.09 |
| Eurasian three-toed woodpecker | Picoides tridactylus | Wo | R | I | C | -0.02 | -1.47 | 1.49 |
| Common buzzard | Buteo buteo | Sev | S | V | OH | 0.04 | -0.08 | 0.16 |
| Eurasian wren | Troglodytes troglodytes | Wo | S | I | G | 0.04 | -0.04 | 0.13 |
| Eurasian magpie | Pica pica | Sev | R | O | OH | 0.05 | -0.06 | 0.16 |
| Carrion crow | Corvus corone | F | R | O | OH | 0.08 | 0.03 | 0.13 |
| Dunnock | Prunella modularis | Wo | S | I | OL | 0.08 | -0.21 | 0.38 |
| Tawny owl | Strix aluco | Wo | R | V | C | 0.1 | -0.35 | 0.54 |
| Common chiffchaff | Phylloscopus collybita | Wo | S | I | G | 0.11 | 0.03 | 0.18 |
| Common starling | Sturnus vulgaris | F | S | I | C | 0.12 | 0.05 | 0.2 |
| Goldcrest | Regulus regulus | Wo | R | I | OH | 0.14 | -0.06 | 0.35 |
| Spotted flycatcher | Muscicapa striata | Sev | L | I | OH | 0.18 | 0.02 | 0.35 |
| Common firecrest | Regulus ignicapilla | Wo | S | I | OH | 0.19 | 0.03 | 0.35 |
| White wagtail | Motacilla alba | F | S | I | N | 0.28 | 0.21 | 0.35 |
| European pied flycatcher | Ficedula hypoleuca | Wo | L | I | C | 0.34 | 0.01 | 0.64 |
| Water pipit | Anthus spinoletta | A | S | I | G | 0.35 | -0.1 | 0.79 |
| Red kite | Milvus milvus | F | S | V | OH | 0.35 | 0.22 | 0.48 |
| Fieldfare | Turdus pilaris | F | S | I | OH | 0.37 | 0.02 | 0.68 |
| Barn swallow | Hirundo rustica | F | L | I | N | 0.38 | 0.28 | 0.48 |
| Italian sparrow | Passer italiae | S | R | P | C | 0.56 | -0.93 | 1.88 |
| Alpine accentor | Prunella collaris | A | R | I | N | 0.61 | 0.05 | 1.18 |
| Hooded crow | Corvus cornix | F | R | O | OH | 1.5 | 0.38 | 2.35 |
| Rock ptarmigan | Lagopus muta | A | R | P | G | 1.73 | 0.79 | 2.66 |
| Alpine chough | Pyrrhocorax graculus | A | R | O | N | - | - | - |
| Black grouse | Lyrurus tetrix | Wo | R | P | G | - | - | - |
| Black redstart | Phoenicurus ochruros | Sev | S | I | N | - | - | - |
| Citril finch | Carduelis citrinella | Wo | S | P | OH | - | - | - |
| Coal tit | Periparus ater | Wo | R | I | C | - | - | - |
| Common cuckoo | Cuculus canorus | Sev | L | I | All | - | - | - |
| Common kestrel | Falco tinnunculus | F | S | V | N | - | - | - |
| Common linnet | Linaria cannabina | F | S | P | OL | - | - | - |
| Common redstart | Phoenicurus phoenicurus | Sev | L | I | C | - | - | - |
| Common rock thrush | Monticola saxatilis | A | L | O | G | - | - | - |
| Common whitethroat | Curruca communis | F | L | I | OL | - | - | - |
| Eurasian chaffinch | Fringilla coelebs | Wo | S | I | OH | - | - | - |
| Eurasian collared dove | Streptopelia decaocto | S | R | P | OH | - | - | - |
| Eurasian coot | Fulica atra | We | S | O | G | - | - | - |
| Eurasian goshawk | Accipiter gentilis | Wo | R | V | OH | - | - | - |
| Eurasian skylark | Alauda arvensis | F | S | I | G | - | - | - |
| Eurasian wryneck | Jynx torquilla | F | L | I | C | - | - | - |
| European goldfinch | Carduelis carduelis | Sev | S | P | OH | - | - | - |
| European robin | Erithacus rubecula | Wo | S | I | OL | - | - | - |
| Grey wagtail | Motacilla cinerea | We | S | I | N | - | - | - |
| Hawfinch | Coccothraustes coccothraustes | Wo | S | P | OH | - | - | - |
| Marsh tit | Poecile palustris | Wo | R | I | C | - | - | - |
| Northern wheatear | Oenthe oenthe | A | L | I | G | - | - | - |
| Red-backed shrike | Lanius collurio | F | L | I | OL | - | - | - |
| Red crossbill | Loxia curvirostra | Wo | S | P | OH | - | - | - |
| Ring ouzel | Turdus torquatus | Wo | S | I | OH | - | - | - |
| Rock bunting | Emberiza cia | Sev | S | I | G | - | - | - |
| Rock partridge | Alectoris graeca | A | R | P | G | - | - | - |
| Song thrush | Turdus philomelos | Wo | S | I | OH | - | - | - |
| Tree pipit | Anthus trivialis | F | L | I | G | - | - | - |
| Wallcreeper | Tichodroma muraria | A | R | I | N | - | - | - |
| Western house martin | Delichon urbicum | S | L | I | N | - | - | - |
| Whinchat | Saxicola rubetra | F | L | I | G | - | - | - |
| White-winged snowfinch | Montifringilla nivalis | A | R | I | N | - | - | - |
| Willow warbler | Phylloscopus trochilus | Wo | L | I | G | - | - | - |
| Yellowhammer | Emberiza citrinella | F | S | P | G | - | - | - |

^a^ Habitat: A = alpine habitat, F = farmland, S = settlement, Sev = several, We = wetland, Wo = woodland

^b^ migration: R = resident, S = short-distance, L= long-distance

^c^ food: O = omnivorous, I = insectivorous, H = herbivorous, V = vertebrates

^d^ nest-site: N = breeding in niches, G = ground breeding, OH = open nests in trees and high bushes, C = cavities; OL = open nests in bushes at low height. For the analyses, ground breeding and open nests in bushes at low height were merged to “ground”, all others to “higher site nesting”.
