# Supplementary material for: Effects of nitrogen deposition on territory numbers of breeding birds
Source: Conserv Biol. 2025 Aug 15;39(6):e70114. doi: 10.1111/cobi.70114 (PMC12658931; doi:10.1111/cobi.70114)
Supplement: Supplementary file 2 — Supporting Information [file COBI-39-e70114-s003.docx]

**S2**. Variables used in the models. Each row in the data contains a value per species and square (ntot) or per square (for all other variables).

| Variable name | Description | Variable type and transformation | Minimum, maximum and median of raw values |
| --- | --- | --- | --- |
| ntot | number of territories of a species | outcome, log(x +1) | 0 / 1–321 / 0–24 |
| Nitro (for non-farmland species) | N^a^ deposition in kg/ha*year | covariate, standardized^b^ | 2.2 / 42.2 / 15.6^c^ |
| Nitro (for farmland species) | N deposition + N application in kg/ha*year | covariate, standardized | 2.2 / 193.6 / 32.7^c^ |
| Elevation | mean altitude (meter above sea level) | covariate, standardized | 193 / 3173 / 1009 |
| Northness | mean north-south index, orientation of the square | covariate, standardized | -0.94 / 0.96 / -0.03 |
| Slope | mean slope of the square [°] | covariate, standardized | 0.1 / 47.6 / 17.4 |
| Arable land | proportion of arable land | covariate, standardized | 0 / 0.97 / 0 |
| Grassland | proportion of grassland | covariate, standardized | 0 / 0.99 / 0.33 |
| Forest | proportion of forest | covariate, standardized | 0 / 0.99 / 0.23 |
| Buildings | proportion of land covered by buildings | covariate, standardized | 0 / 0.42 / 0 |
| Structures | proportion of structure-rich area like golf parks, parks, hedgerows, bosk, etc. | covariate, standardized | 0 / 0.8 / 0.03 |
| Rivers | length of all watercourses [m] | covariate, standardized | 0 / 8585 / 1749 |
| Roads | length of all roads [m] | covariate, standardized | 0 / 26938 / 2096 |
| Shoreline | occurrence of shoreline | factor (binarized 0/1) | 0 / 1 / 0 |
| Rocks | occurrence of rock or scree | factor (binarized 0/1) | 0 / 1 / 0 |
| Wetlands | occurrence of wetlands | factor (binarized 0/1) | 0 / 1 / 0 |
| Year | year of data sampling | random factor | 2012–2016 |

^a^ N = nitrogen

^b^ “standardized” = centered and scaled to one standard deviation.

^c^ Minimum, maximum and median for ntot gives the range across all species. “nitro” includes N deposition and, for farmland species, N application (manure, fertilizer).
