# Supplementary material for: Effects of nitrogen deposition on territory numbers of breeding birds
Source: Conserv Biol. 2025 Aug 15;39(6):e70114. doi: 10.1111/cobi.70114 (PMC12658931; doi:10.1111/cobi.70114)

**S3.** Effect plots of nitrogen deposition on territory density for each of the 112 studied species. Curves show regression lines for each elevational range, spanning only across N-values observed at each range, with 95% uncertainty intervals (dotted lines). Small dots on each regression line indicate the 15% and 85% quantile value of the N values per elevation range; for a linear relationship, we used the line through these points and averaged it over the elevation ranges.


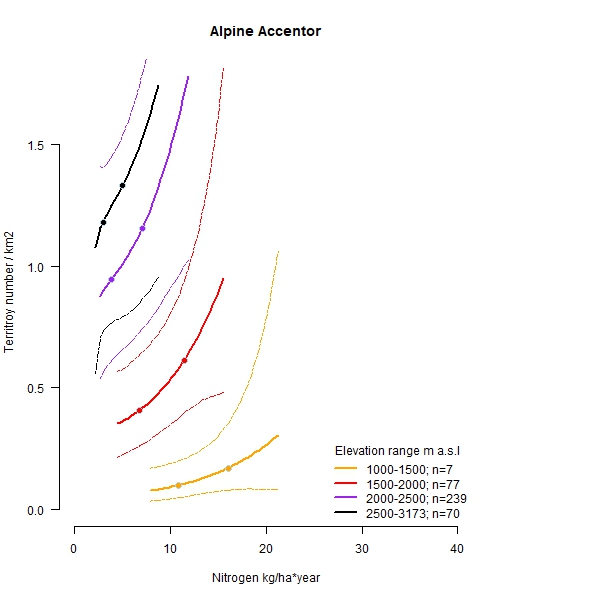

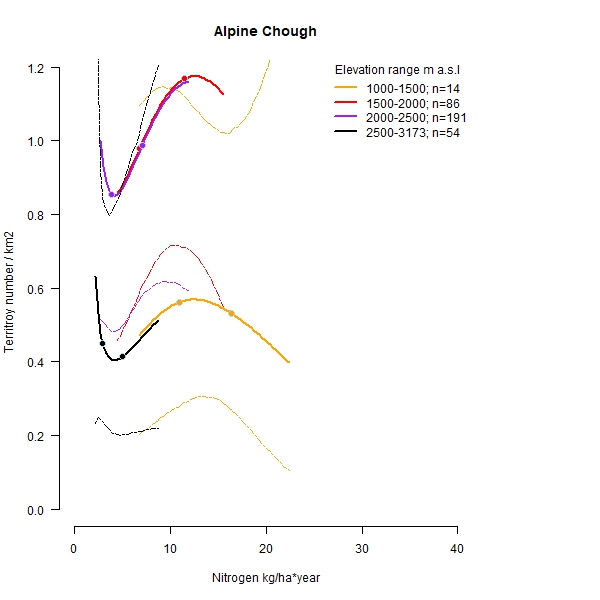

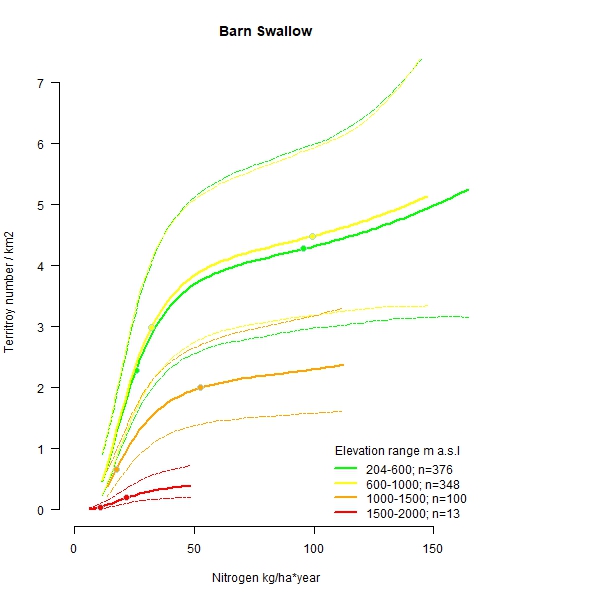

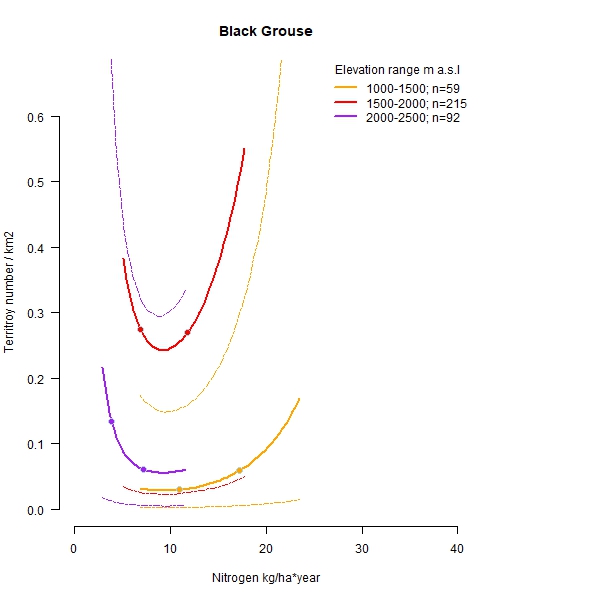

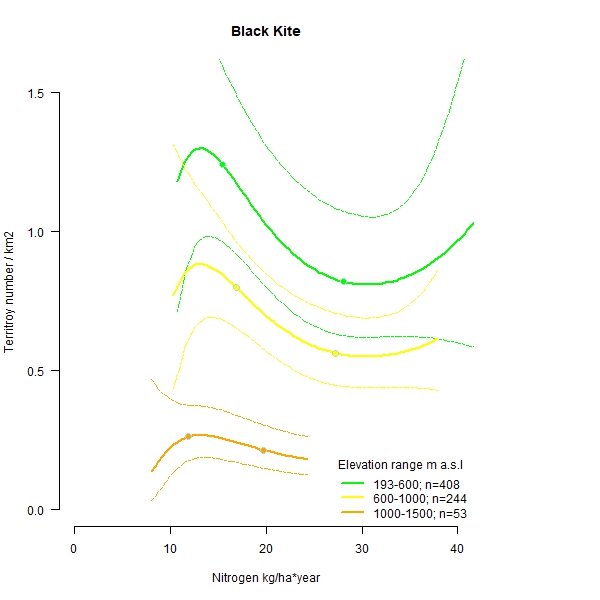

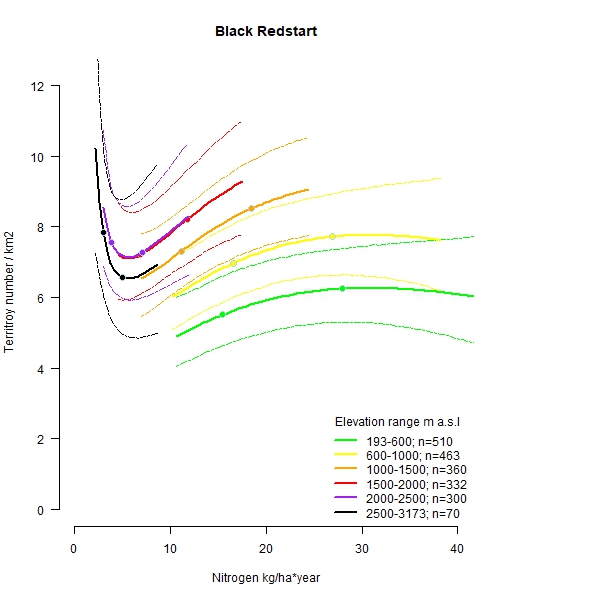

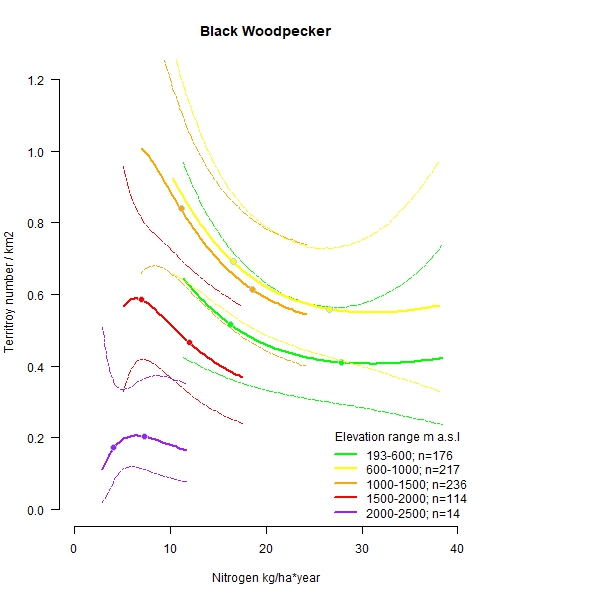

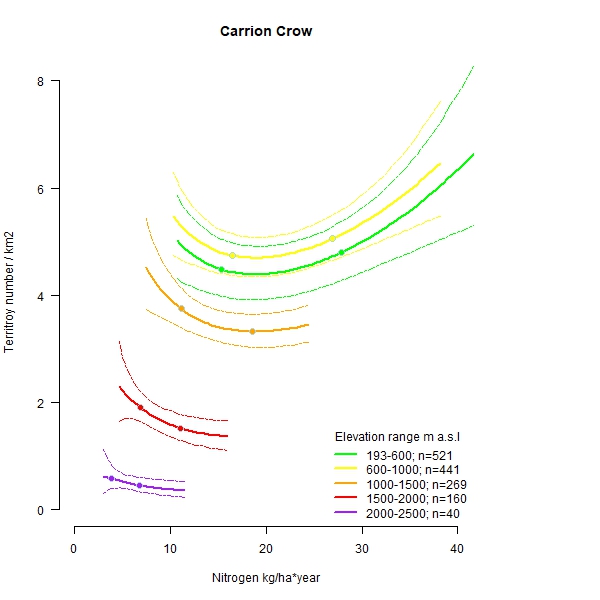

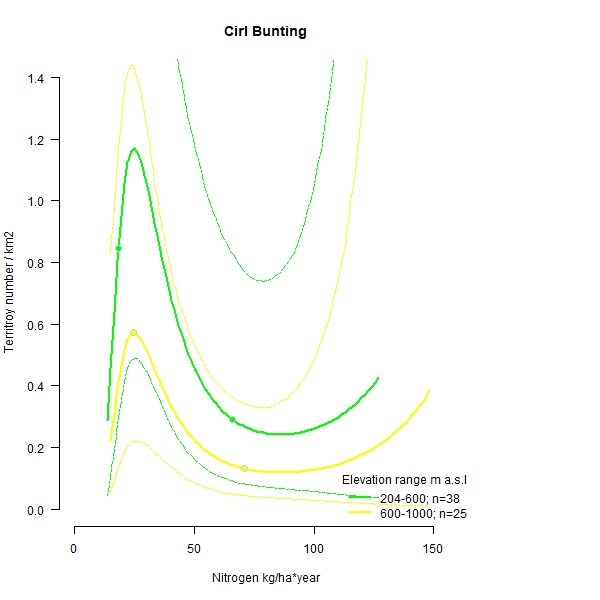

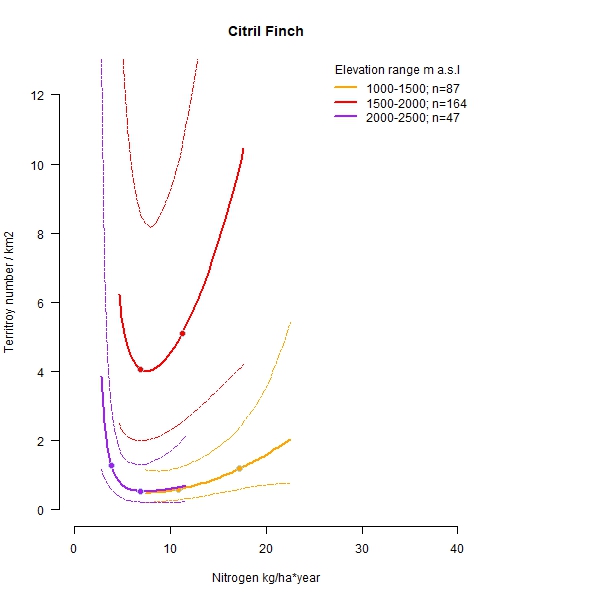

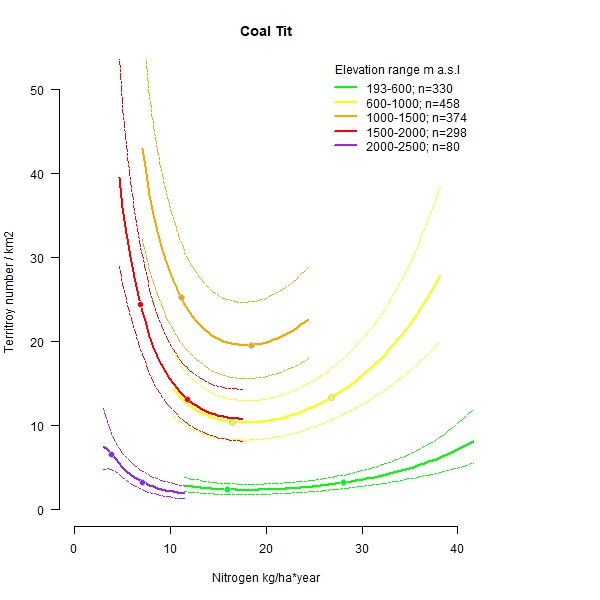

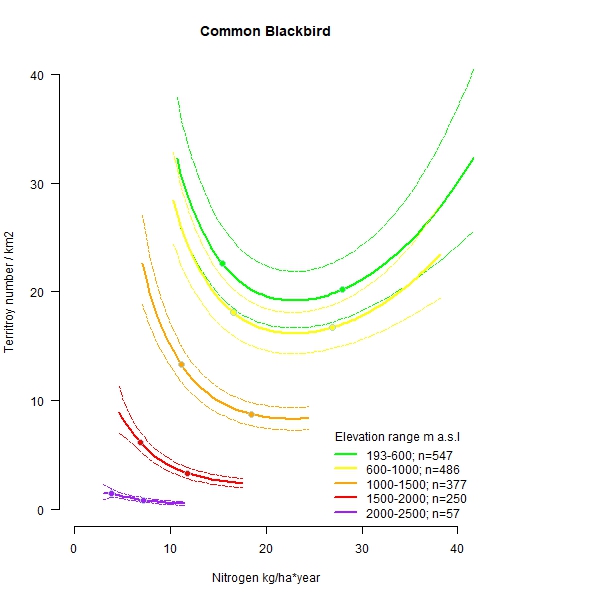

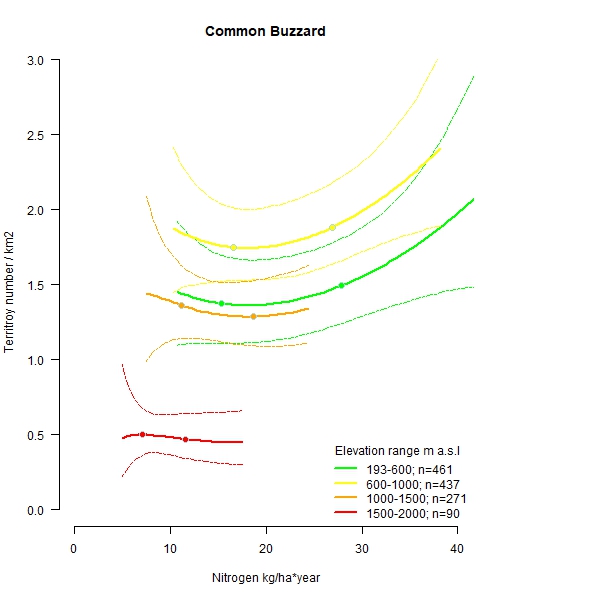

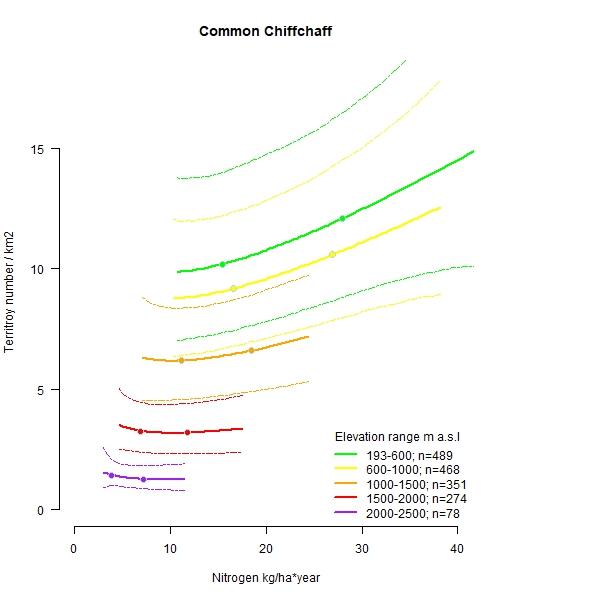

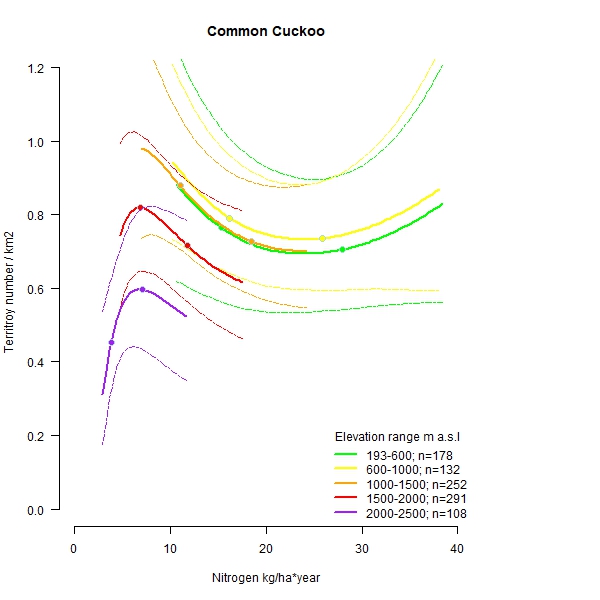

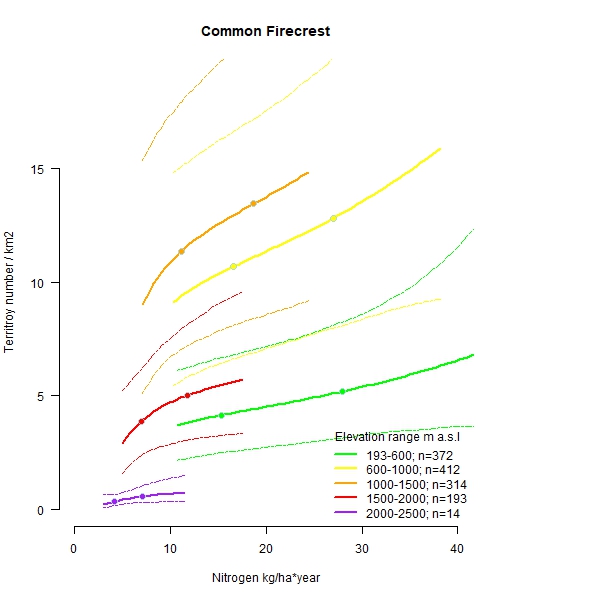

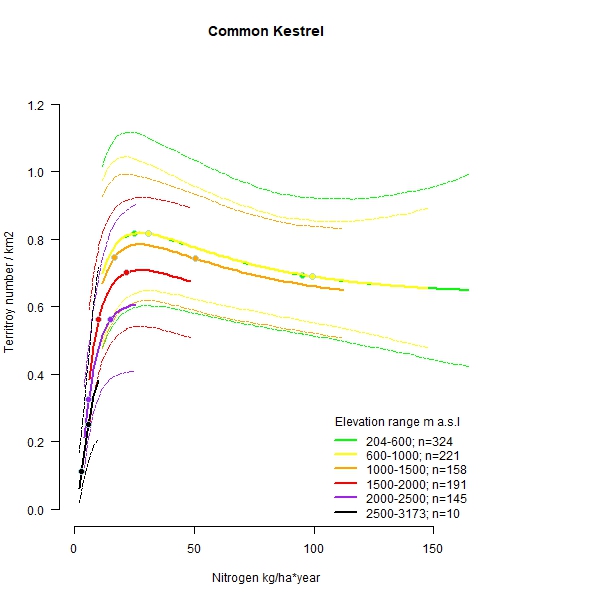

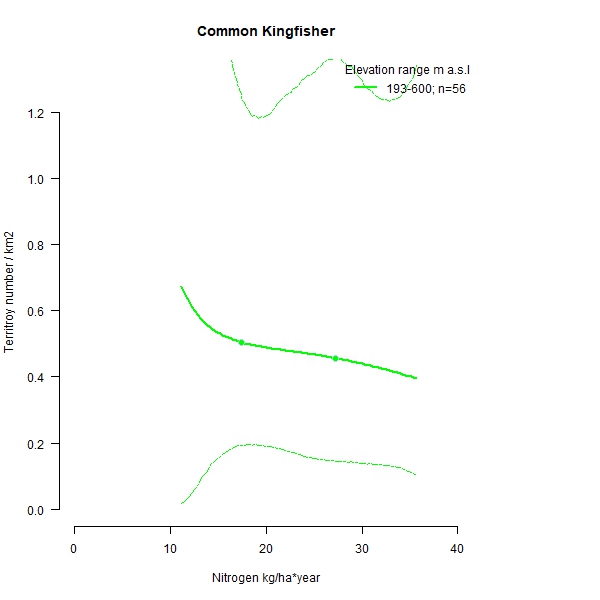

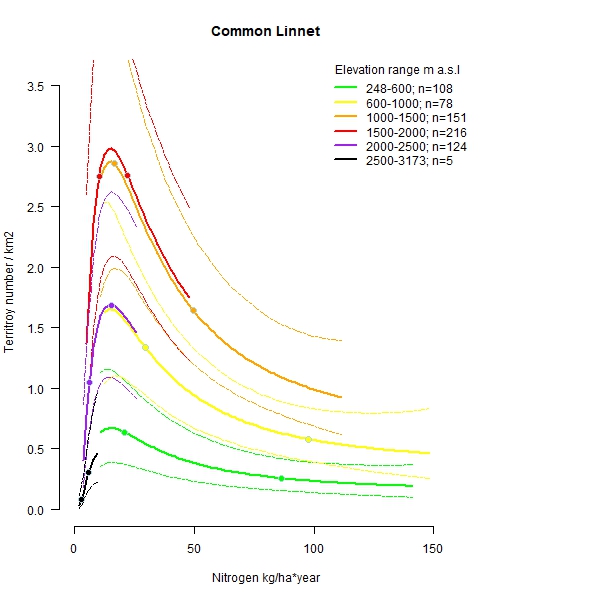

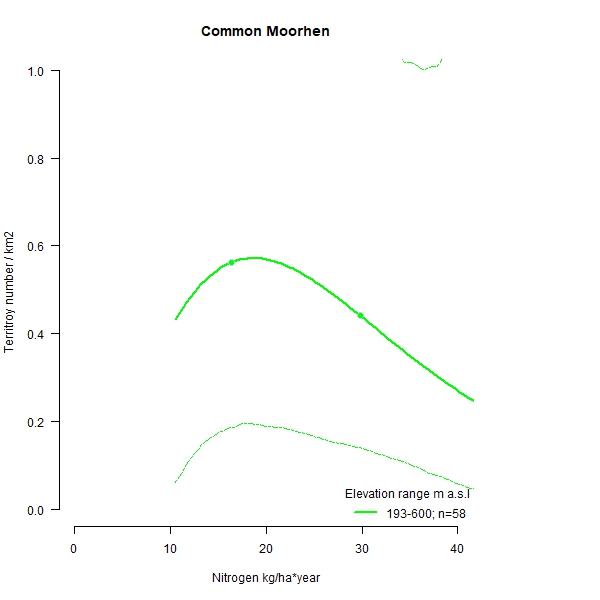

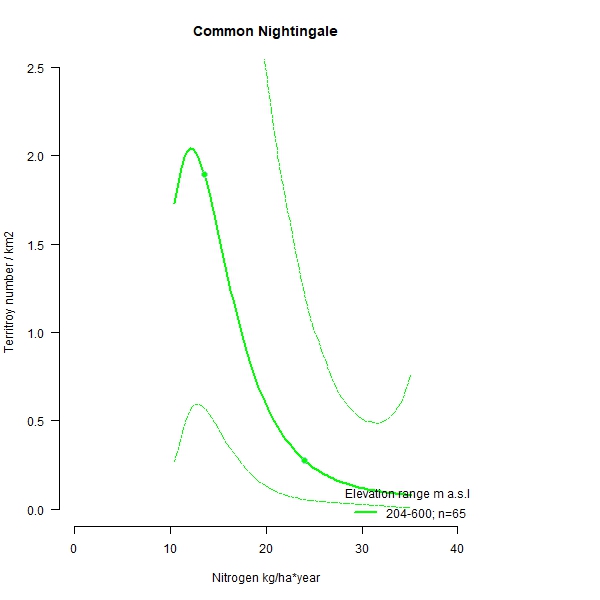

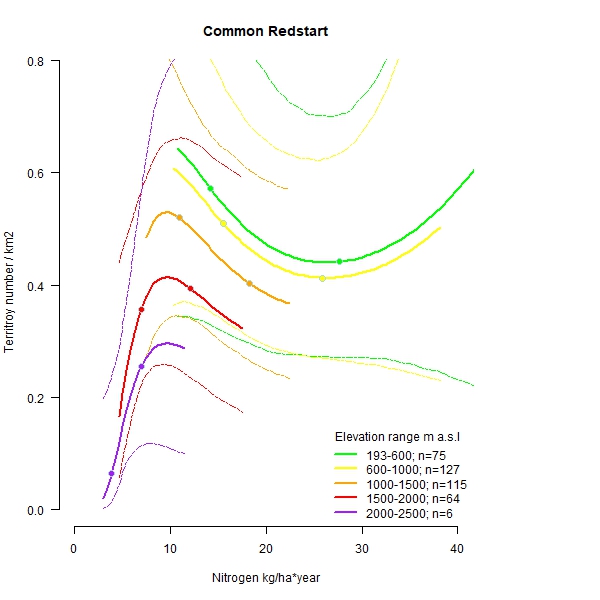

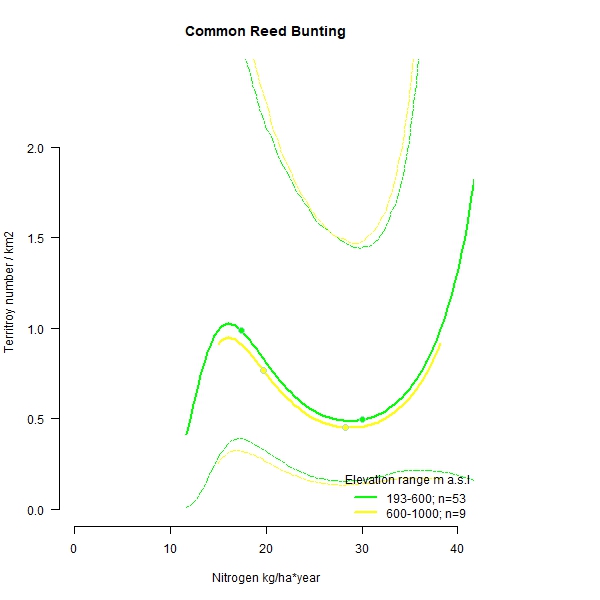

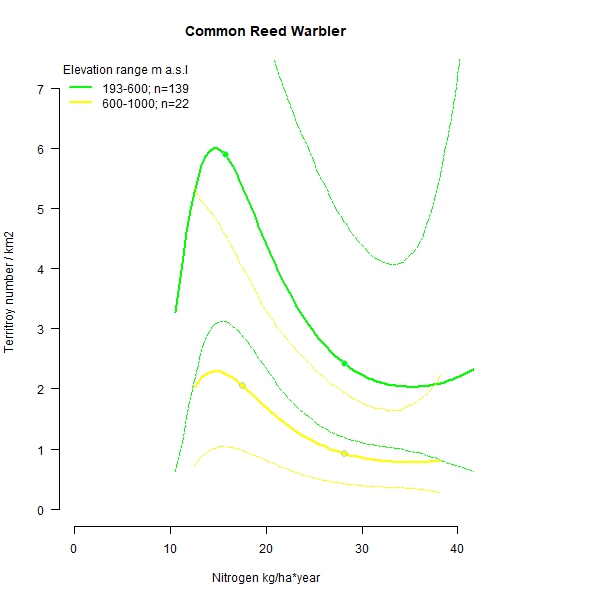

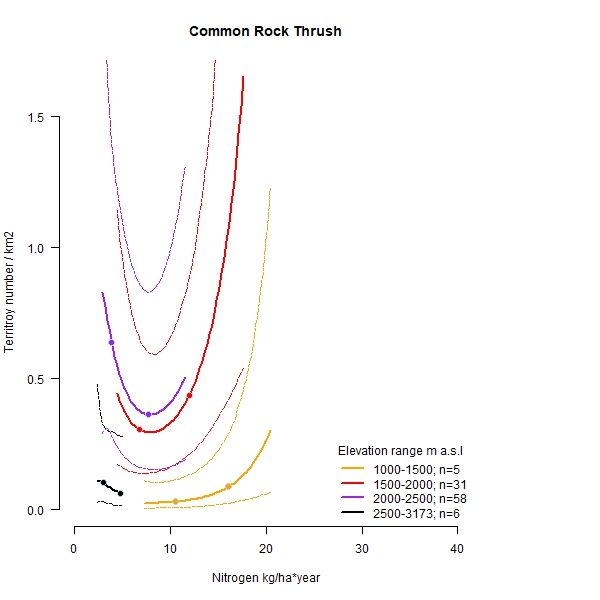

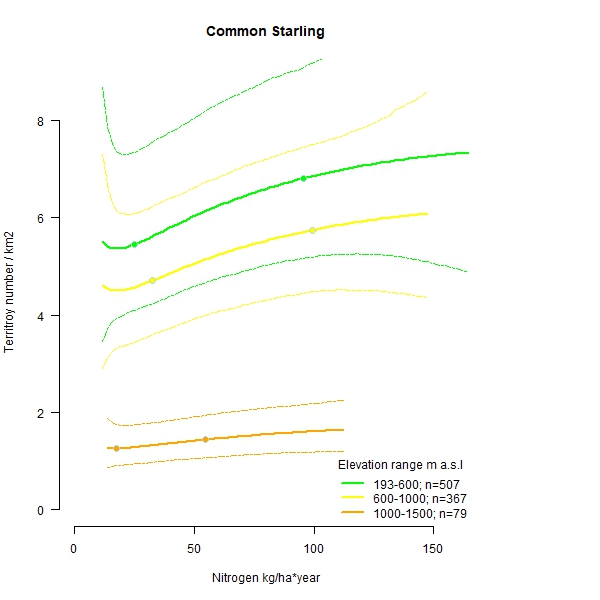

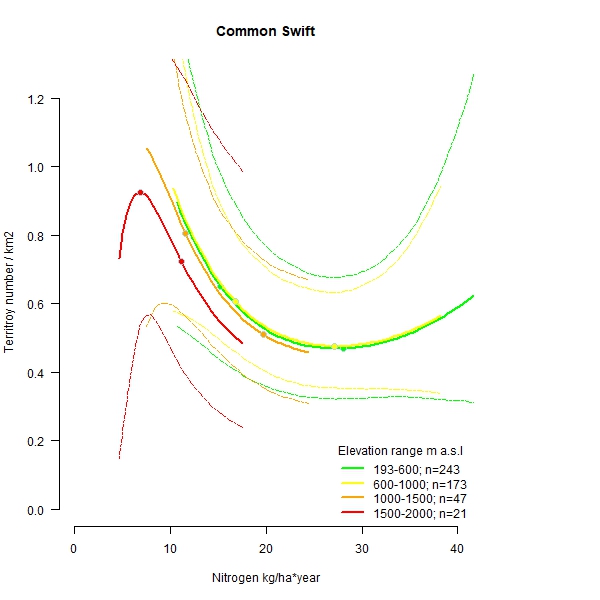

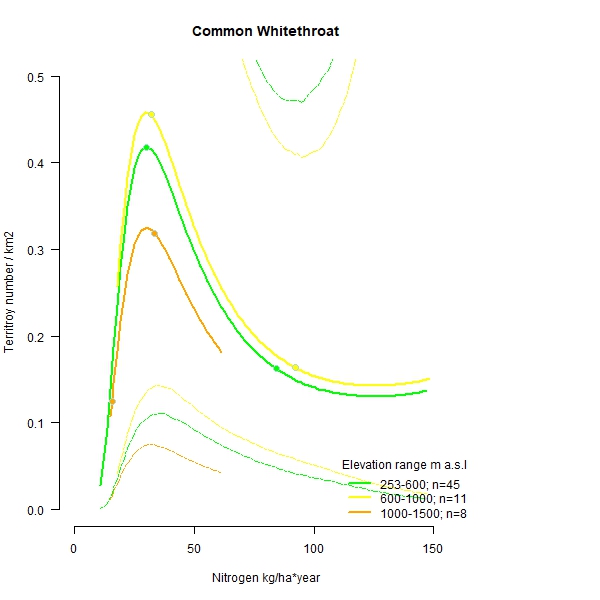

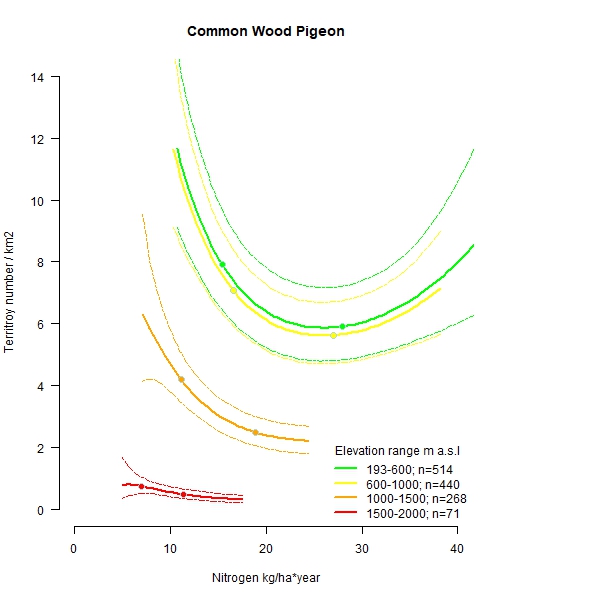

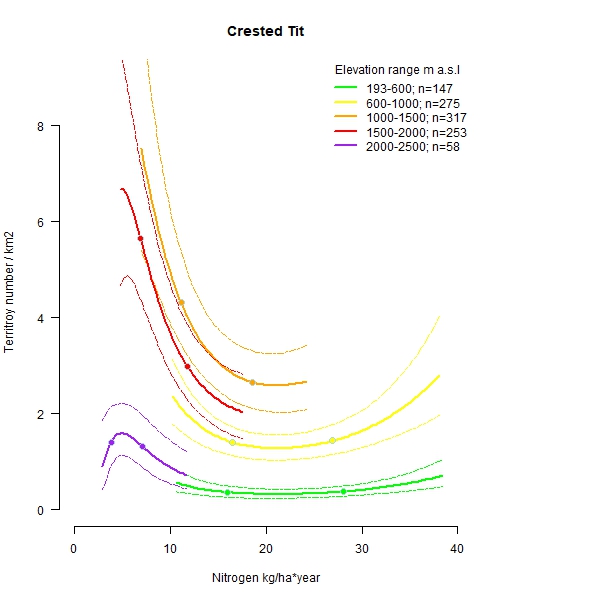

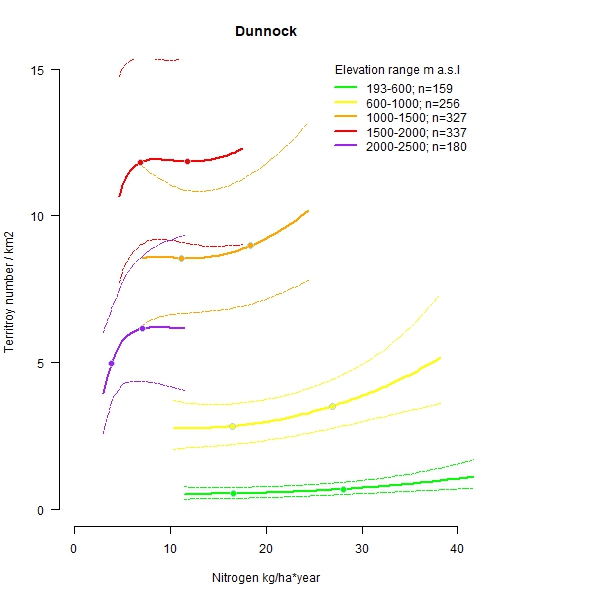

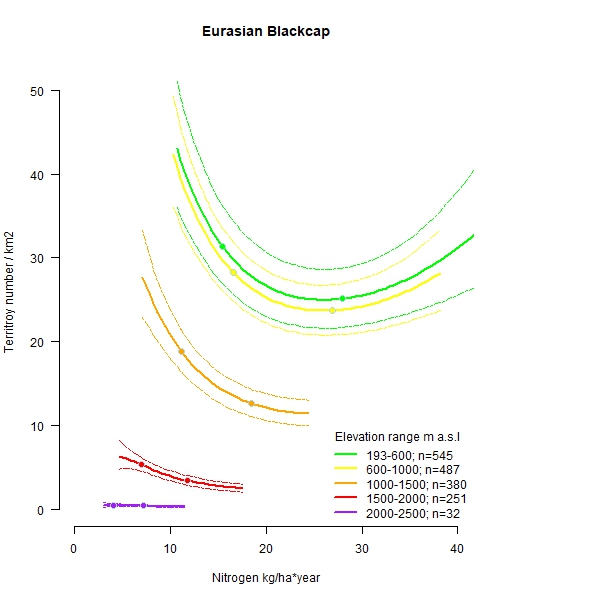

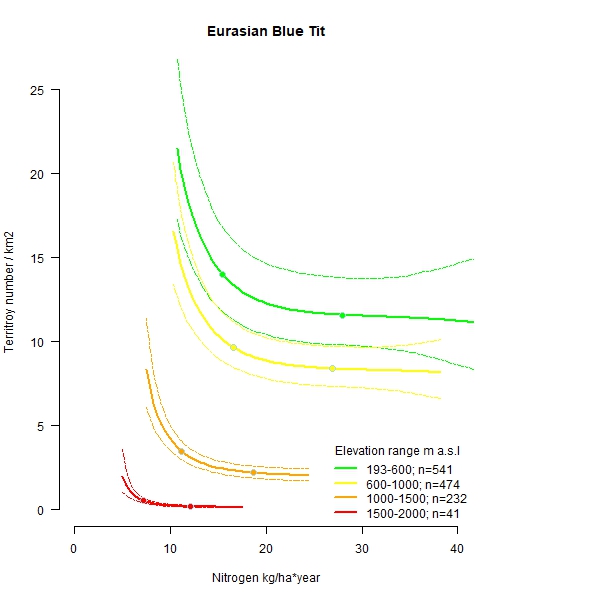

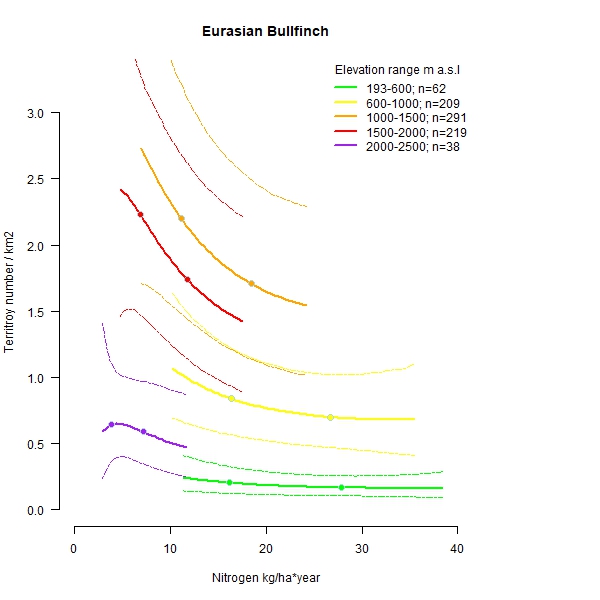

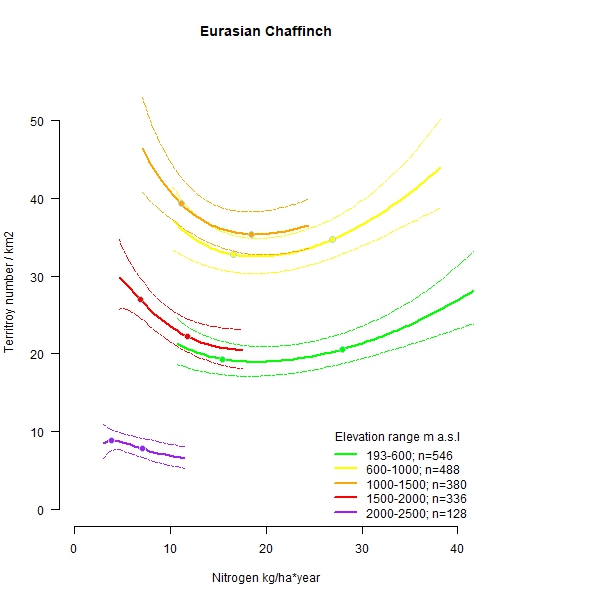

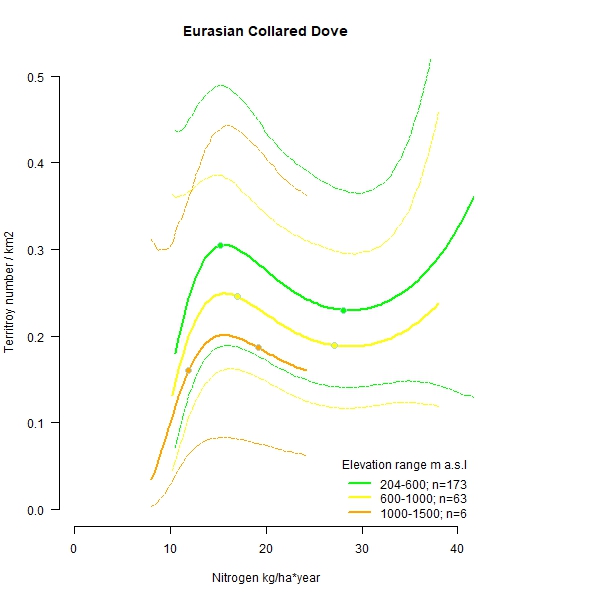

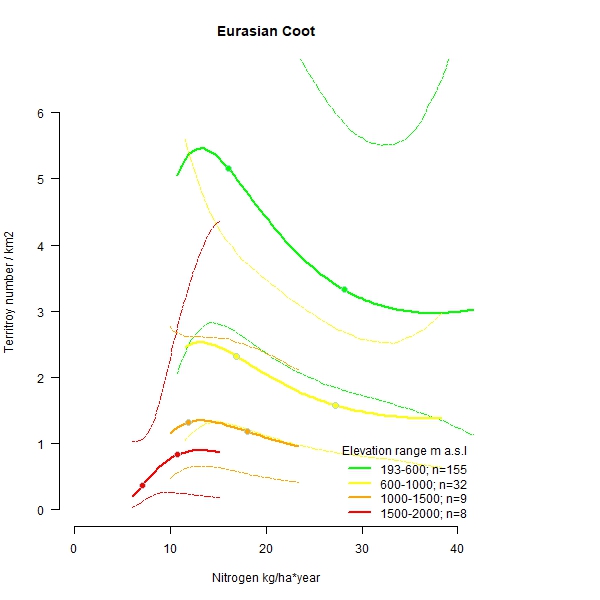

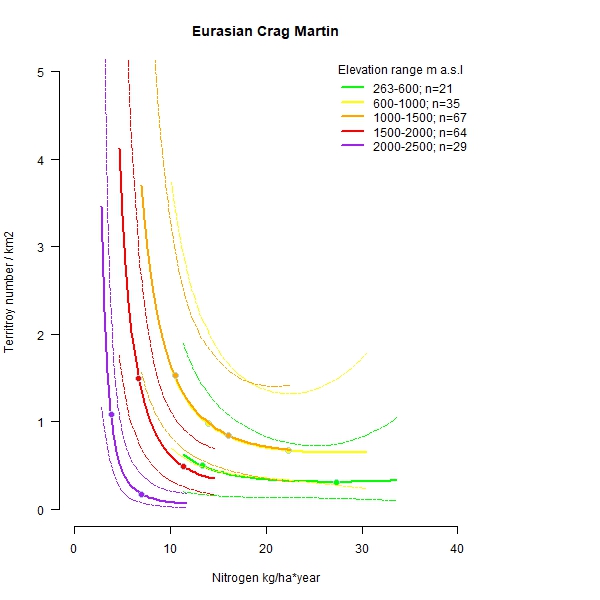

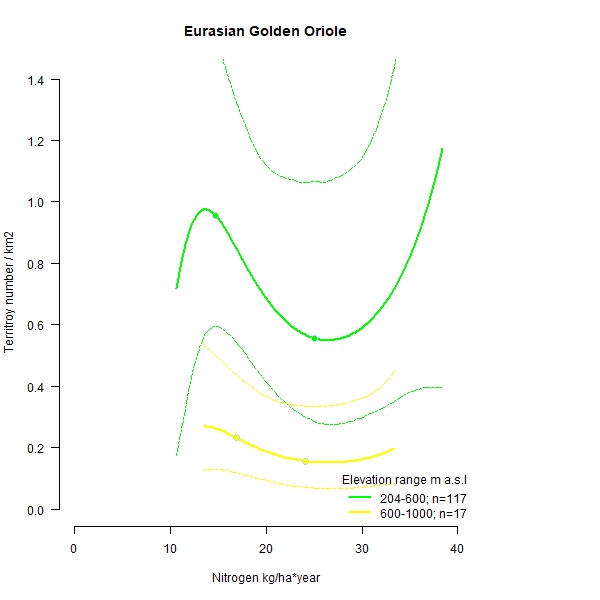

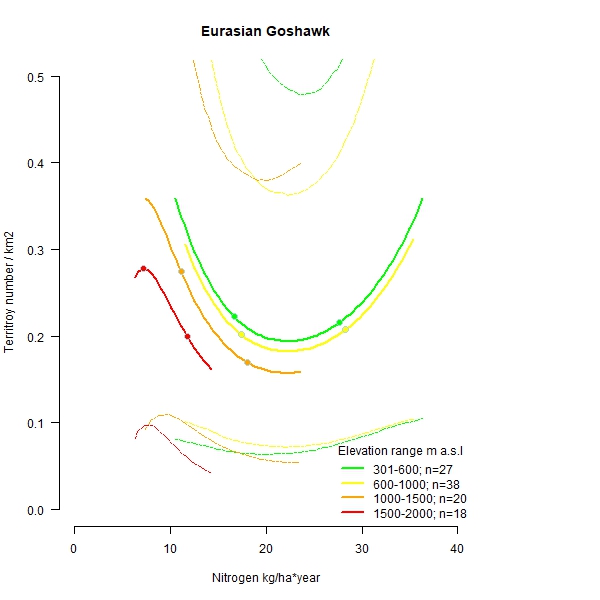

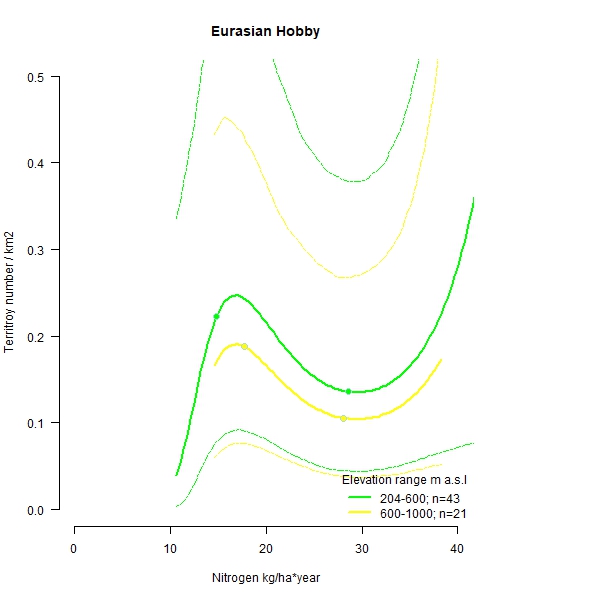

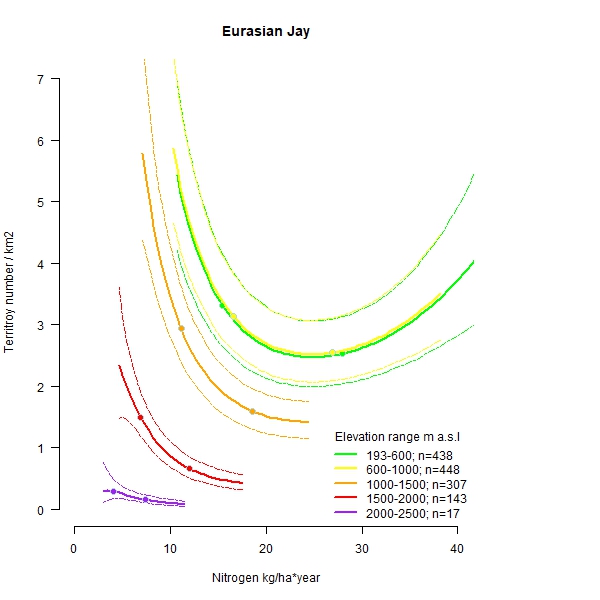

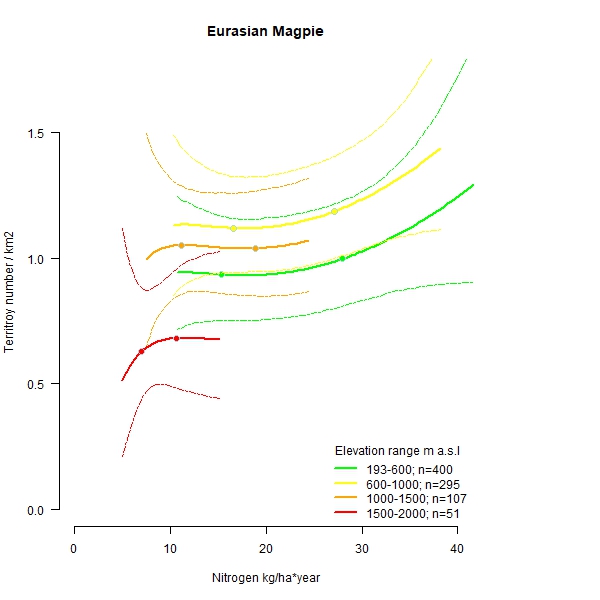

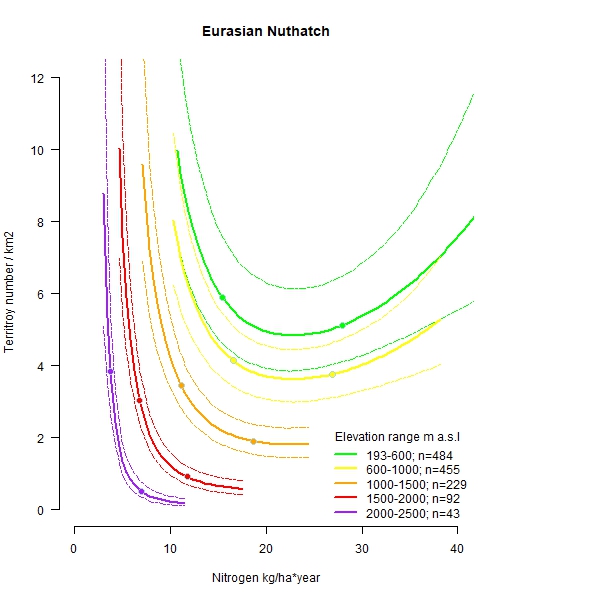

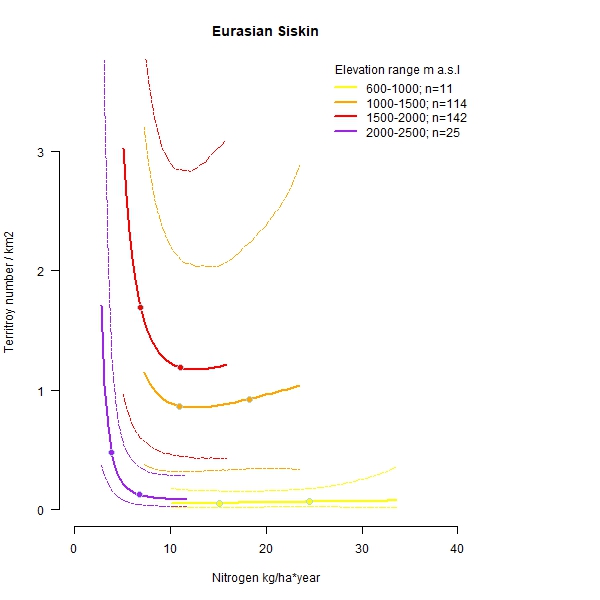

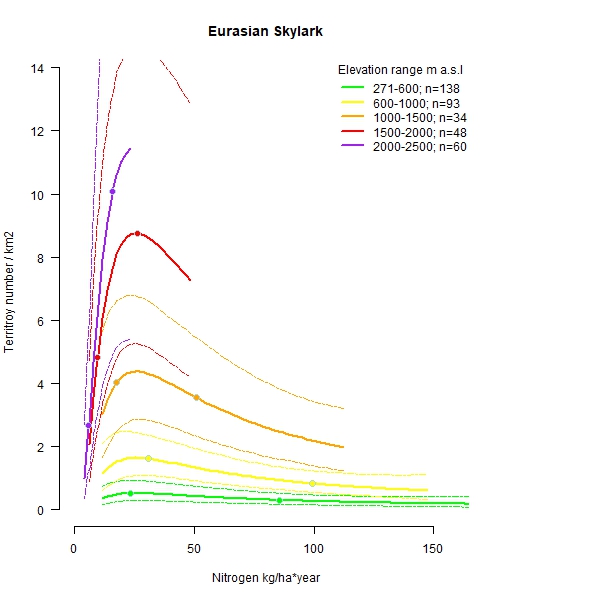

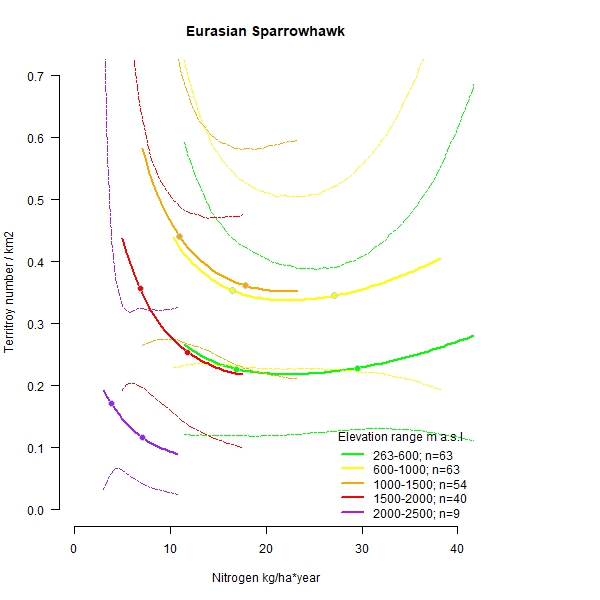

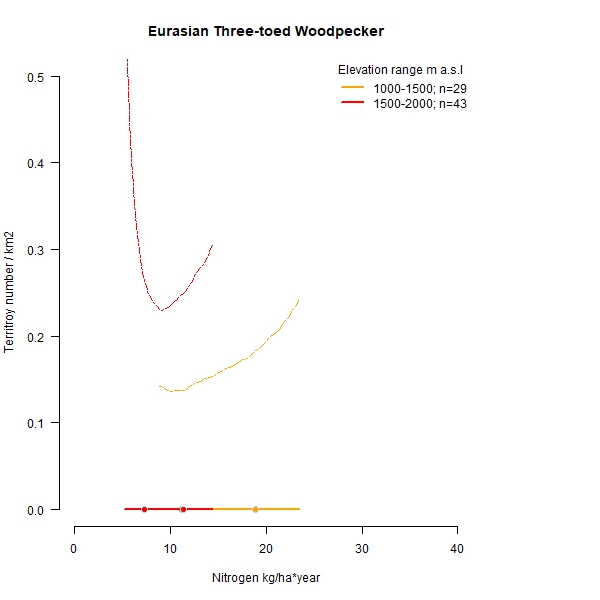

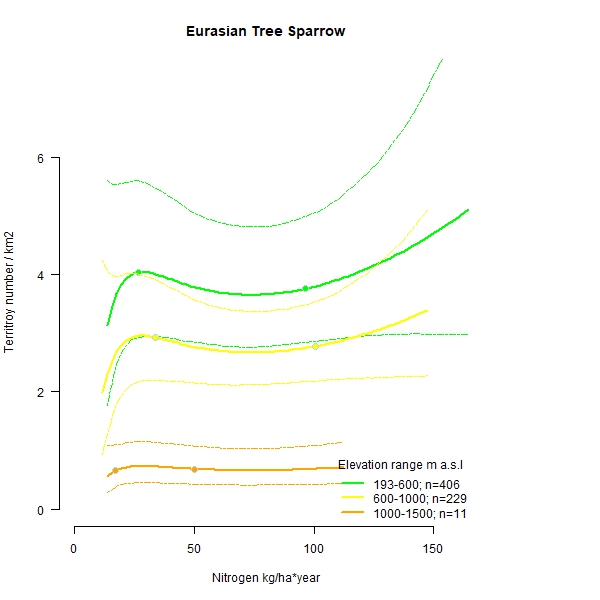

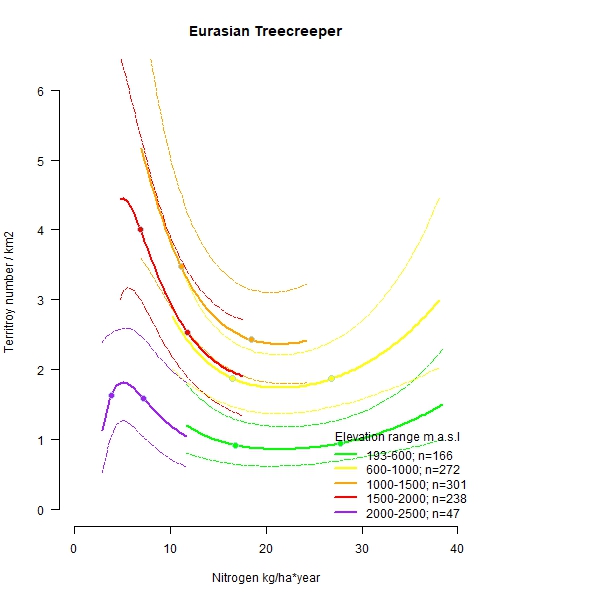

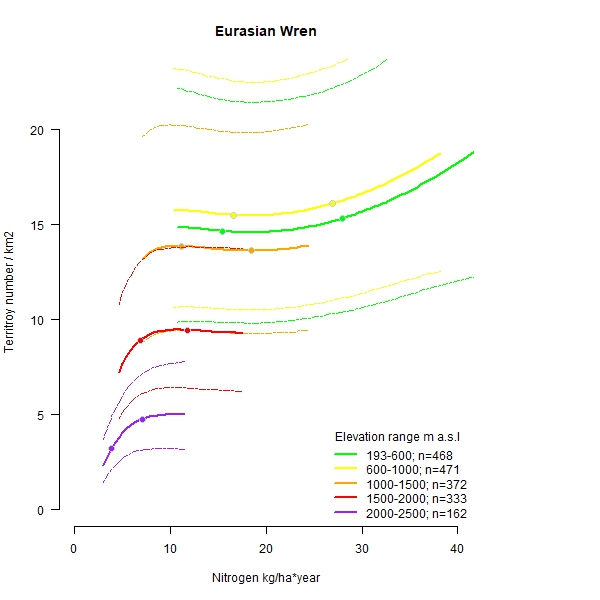

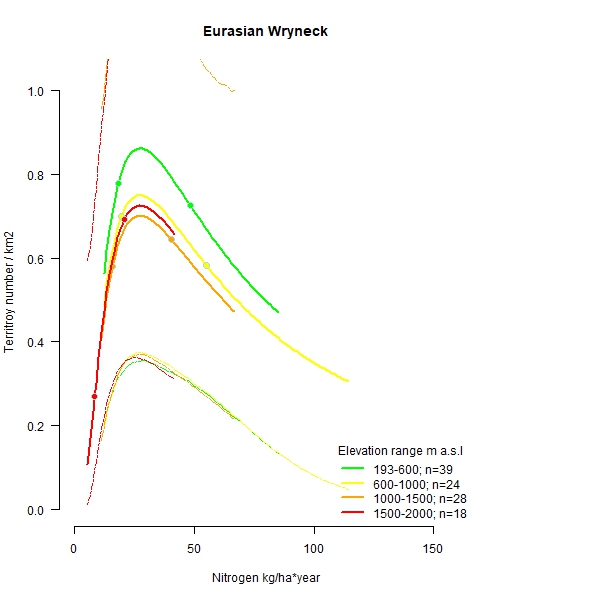

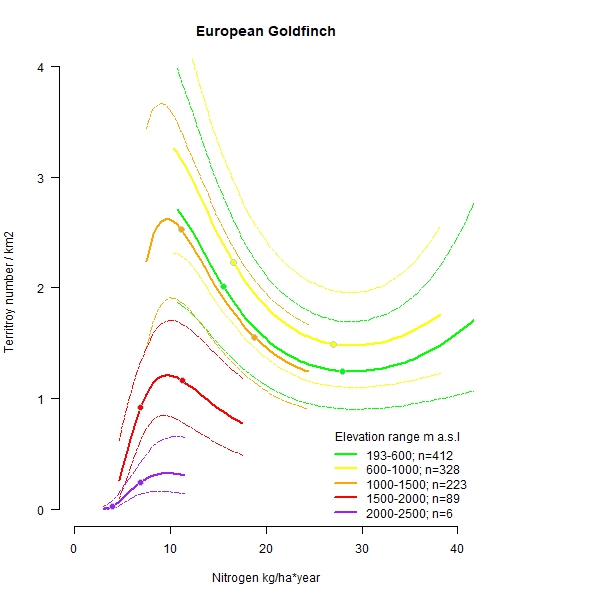

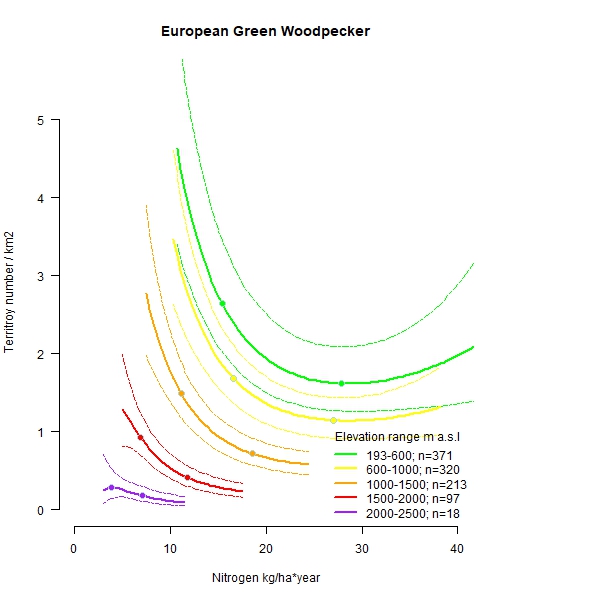

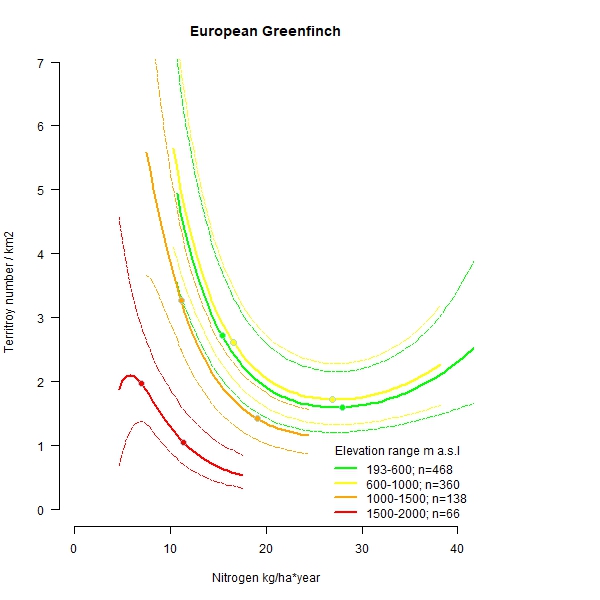

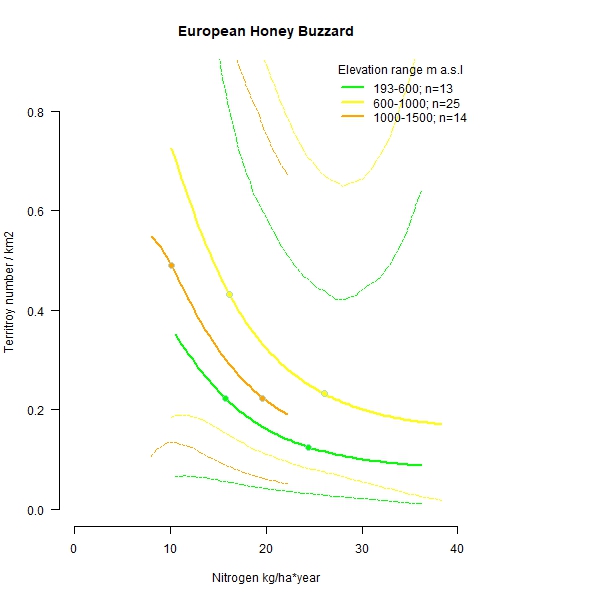

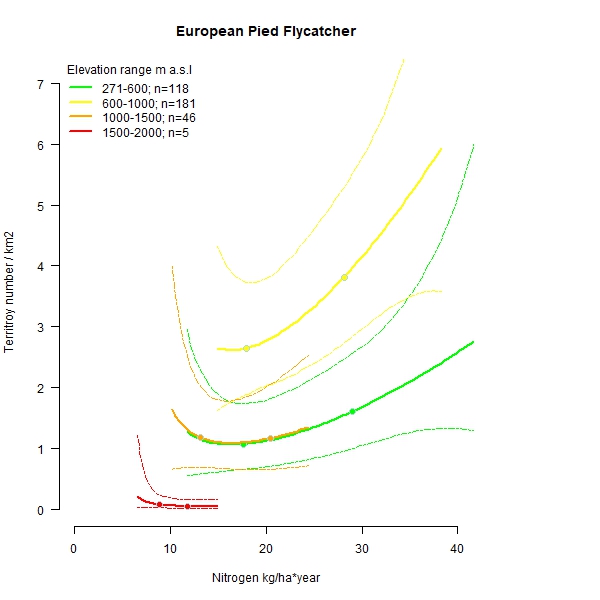

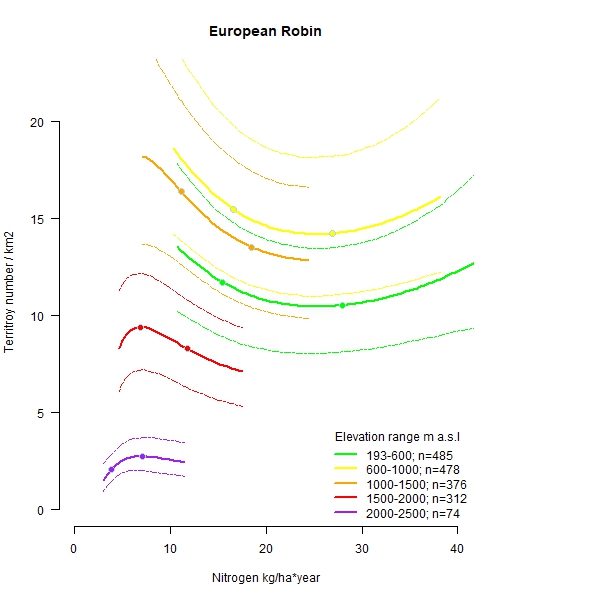

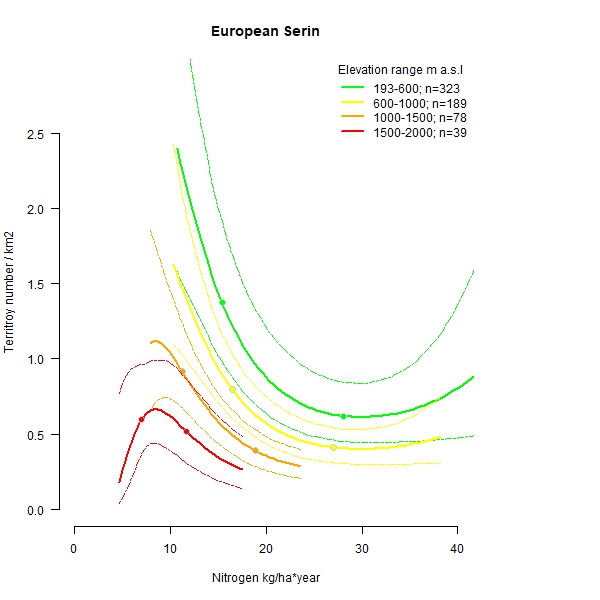

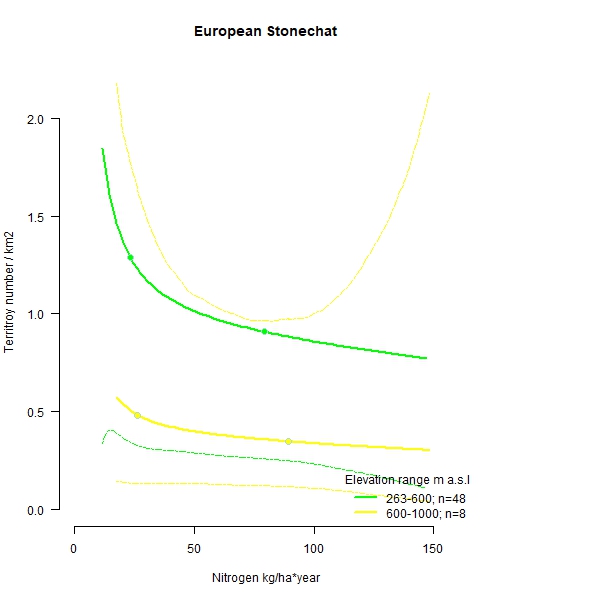

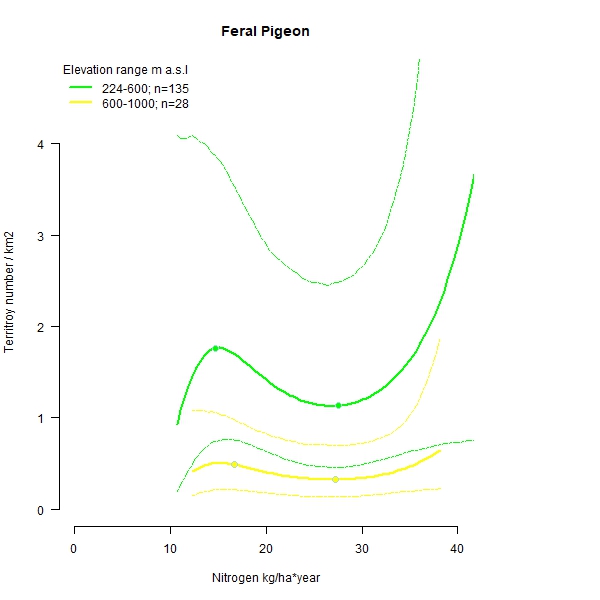

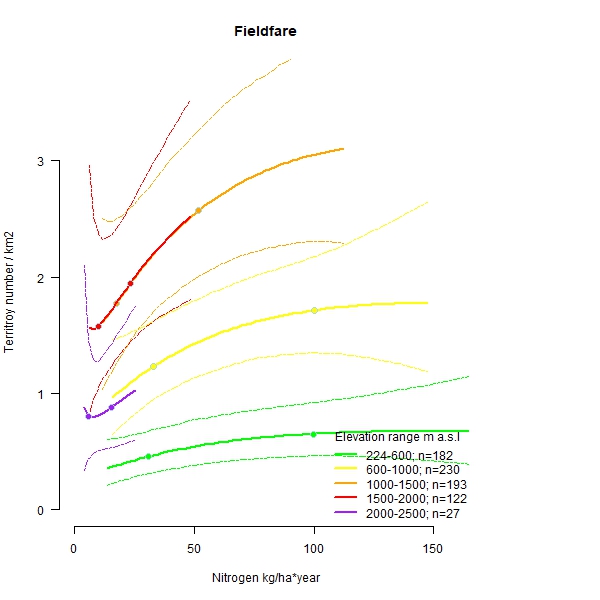

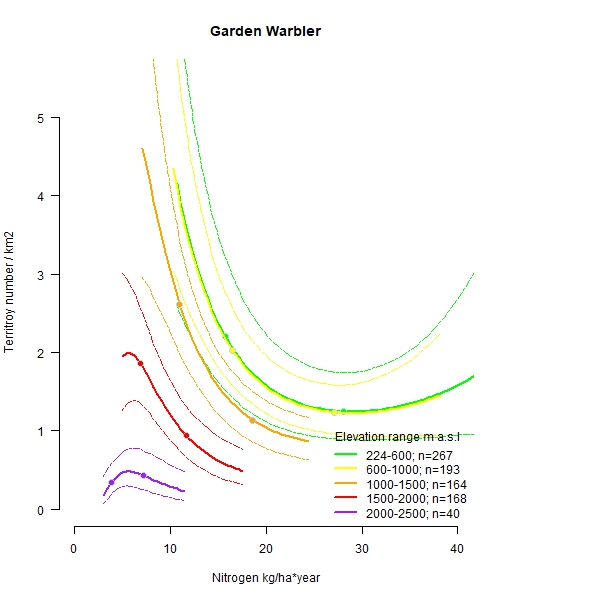

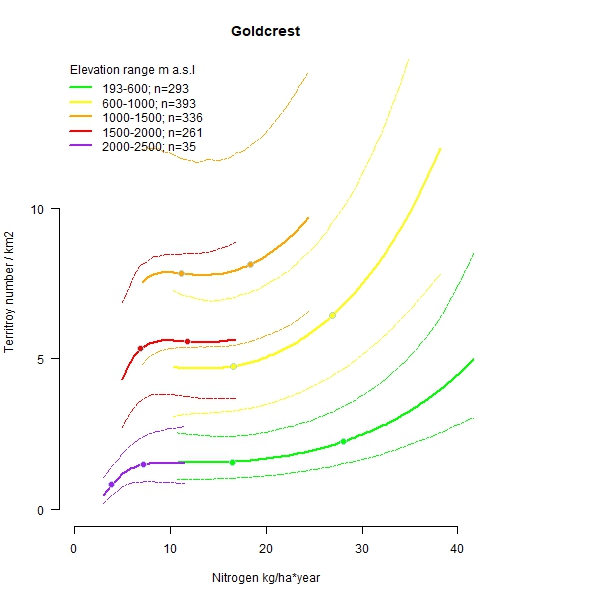

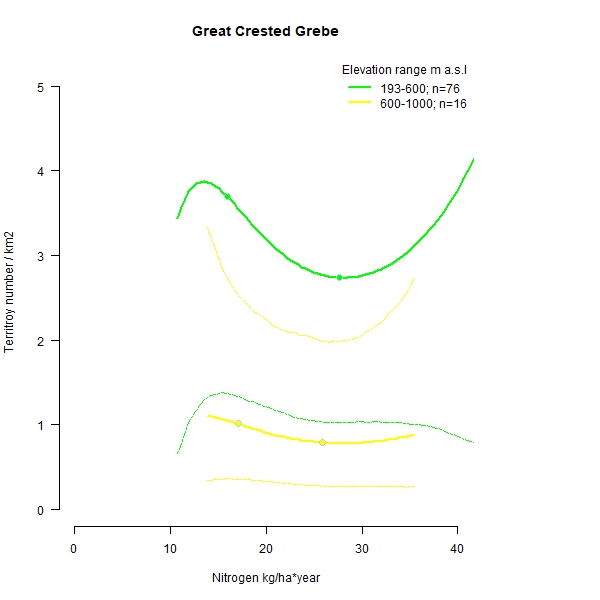

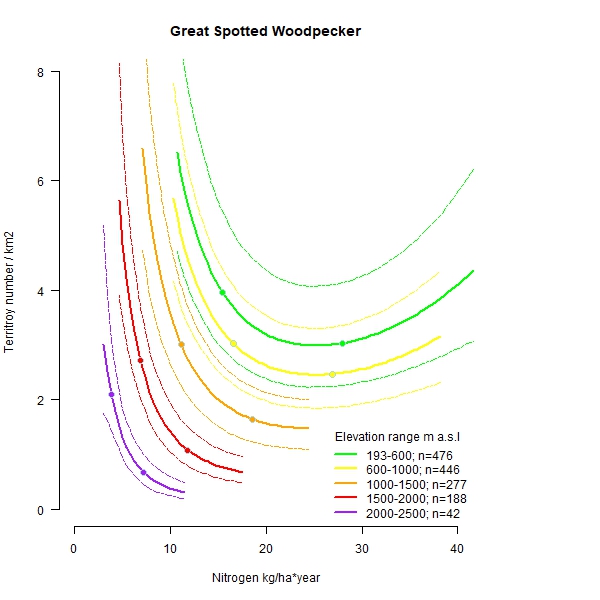

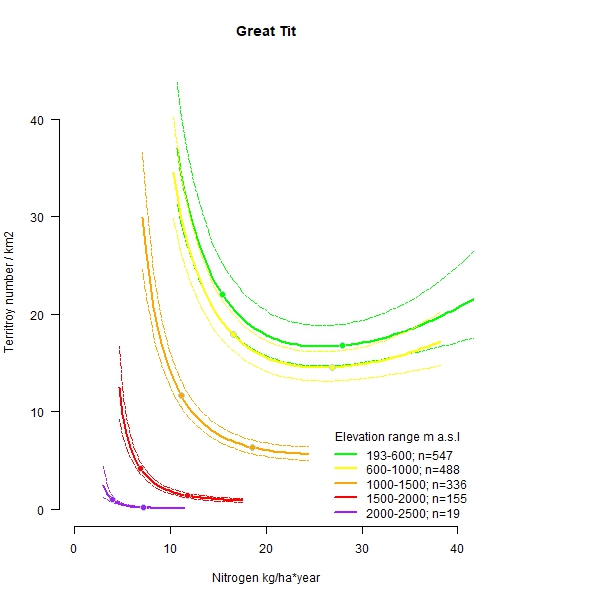

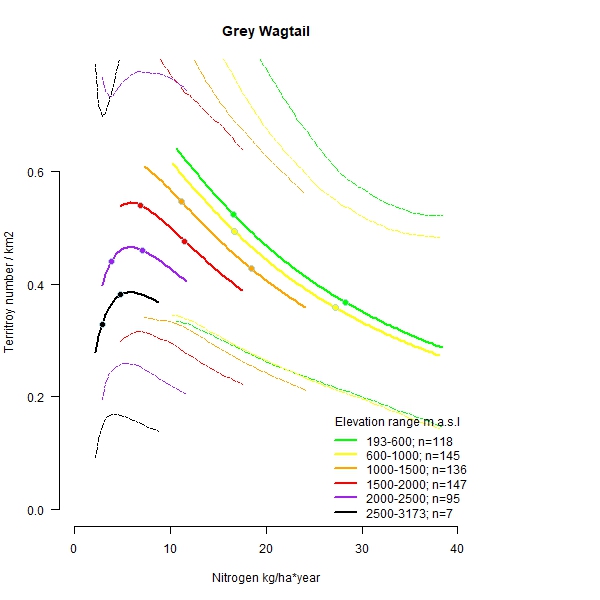

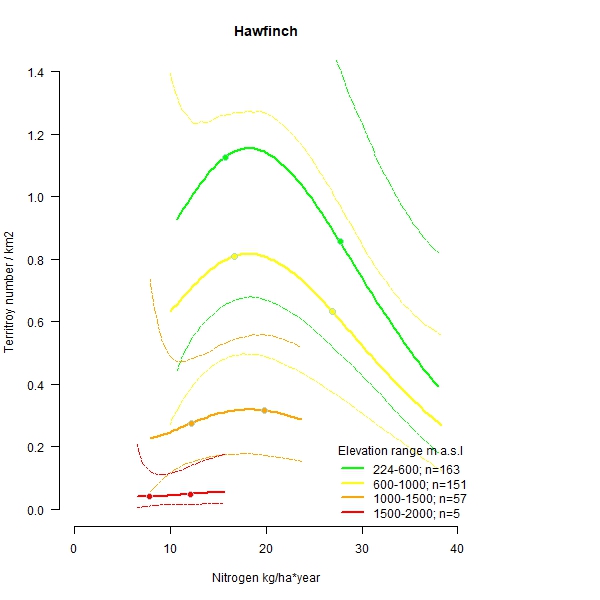

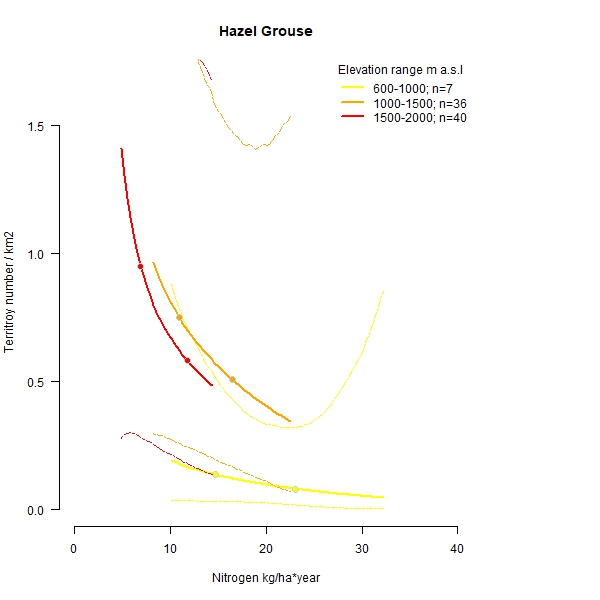

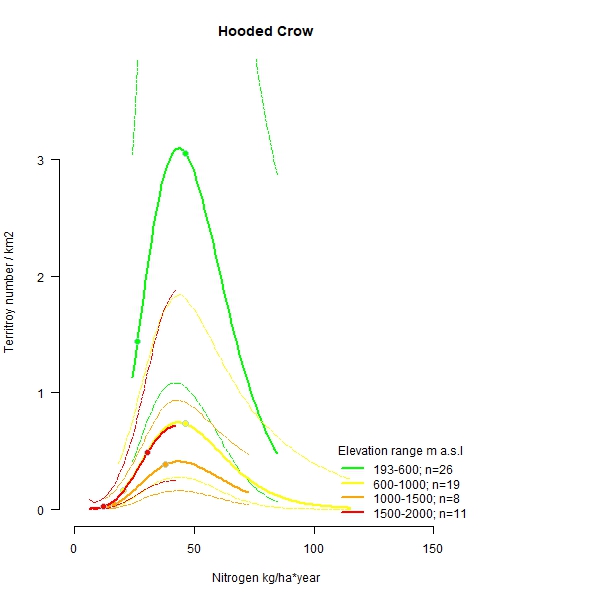

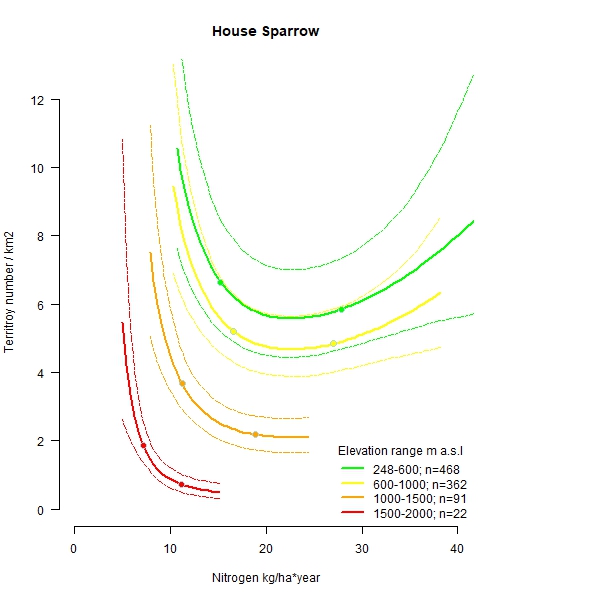

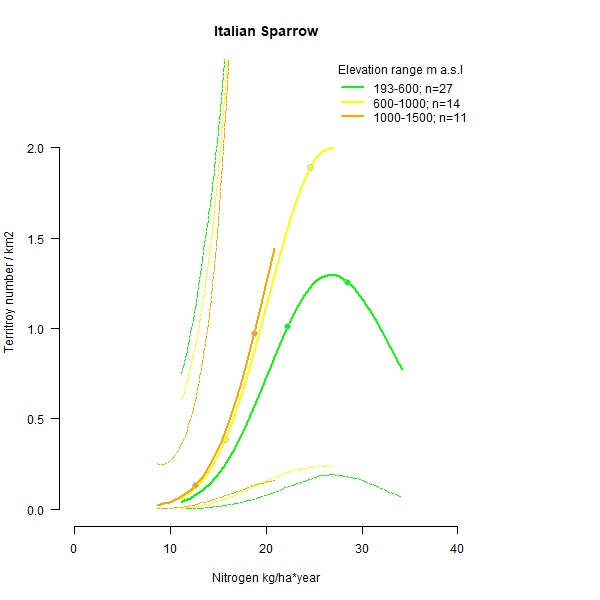

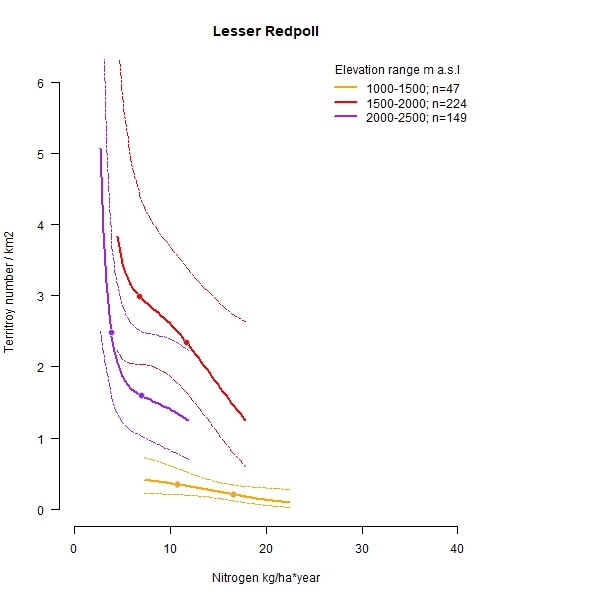

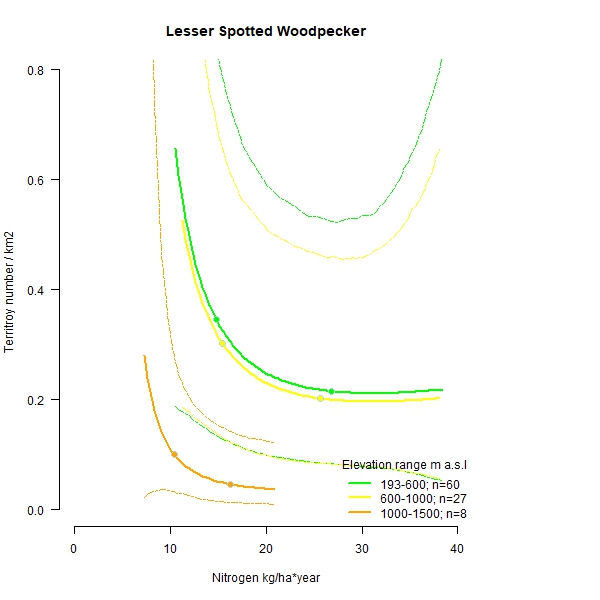

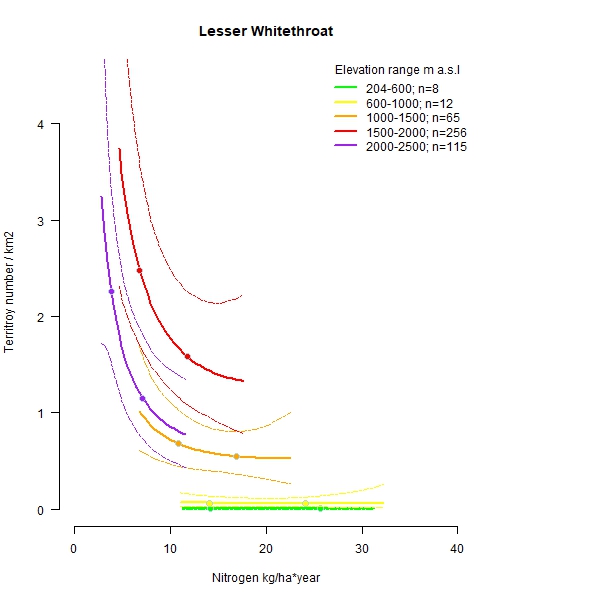

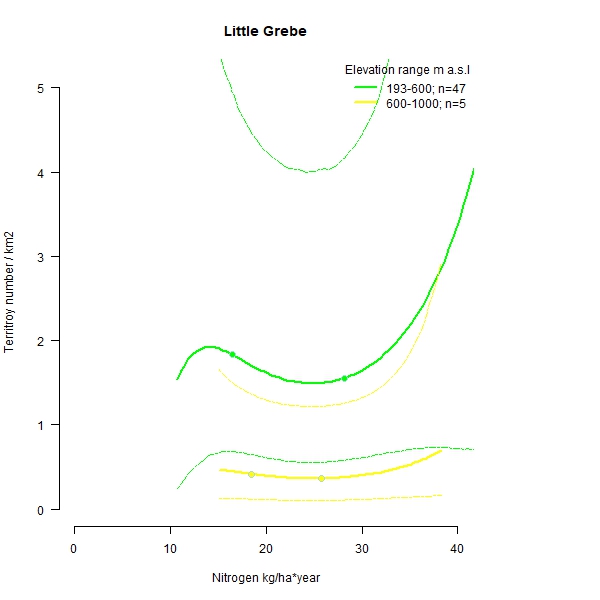

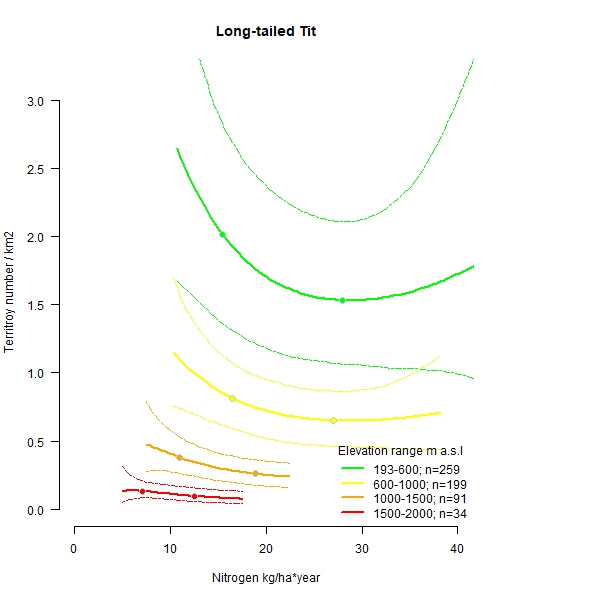

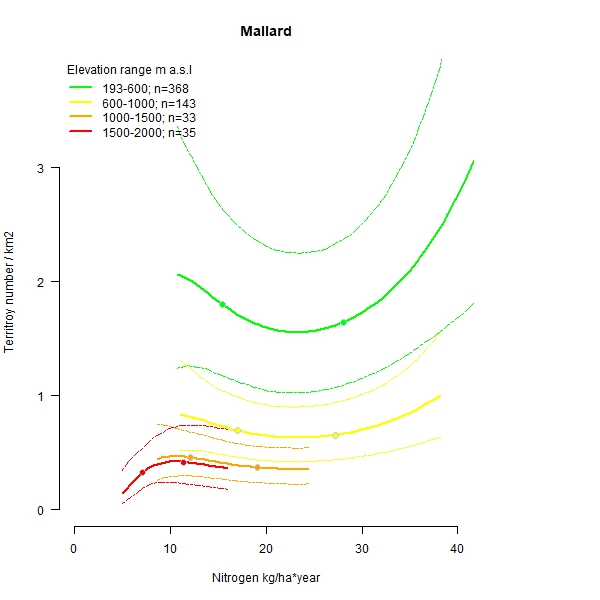

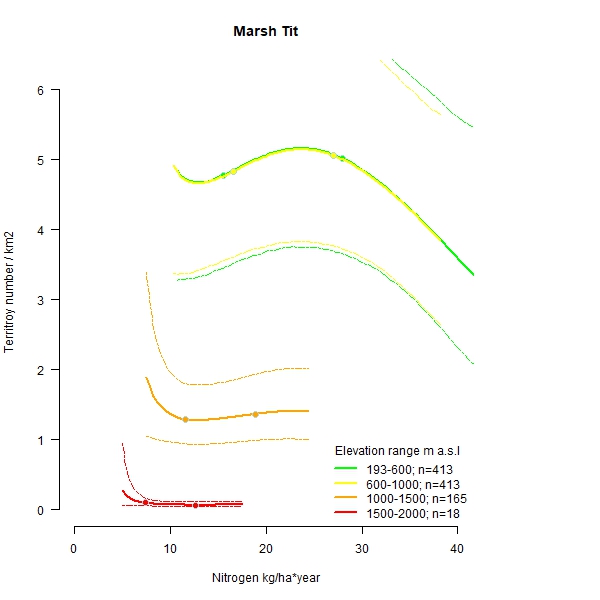

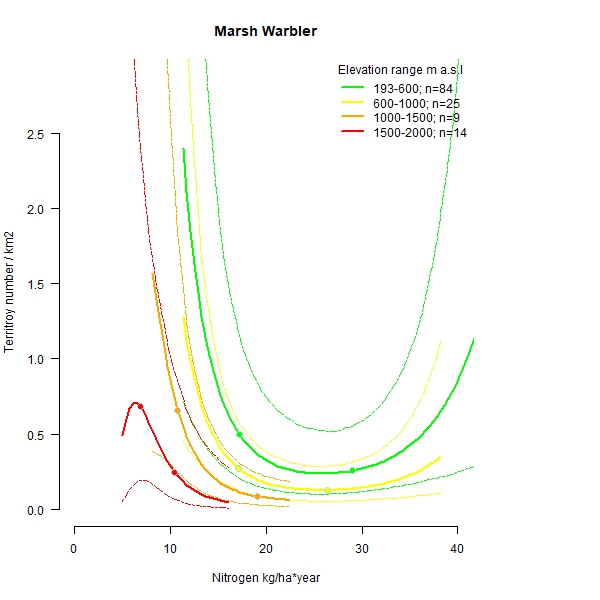

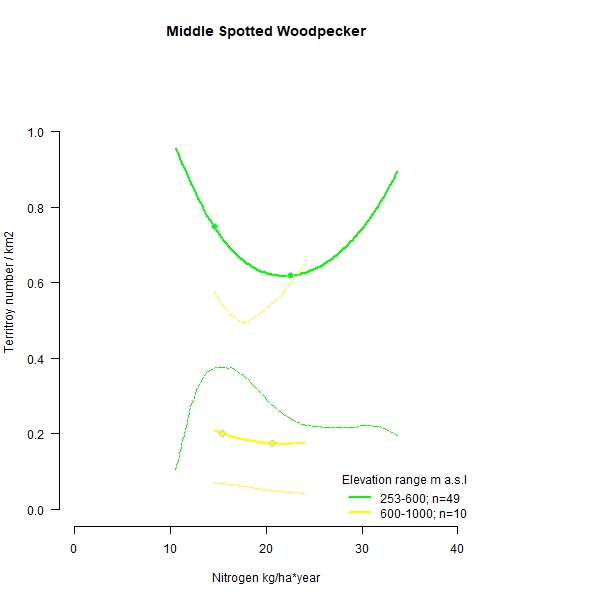

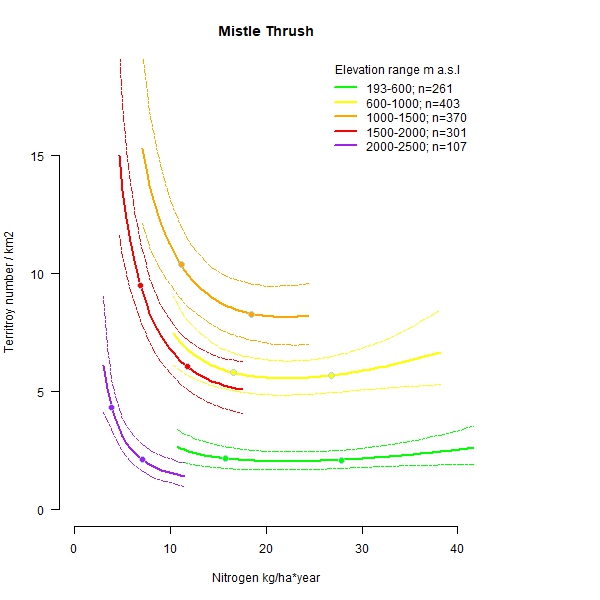

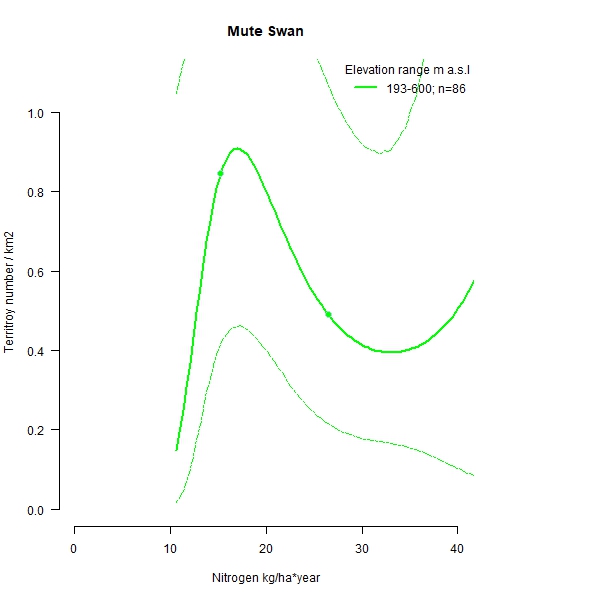

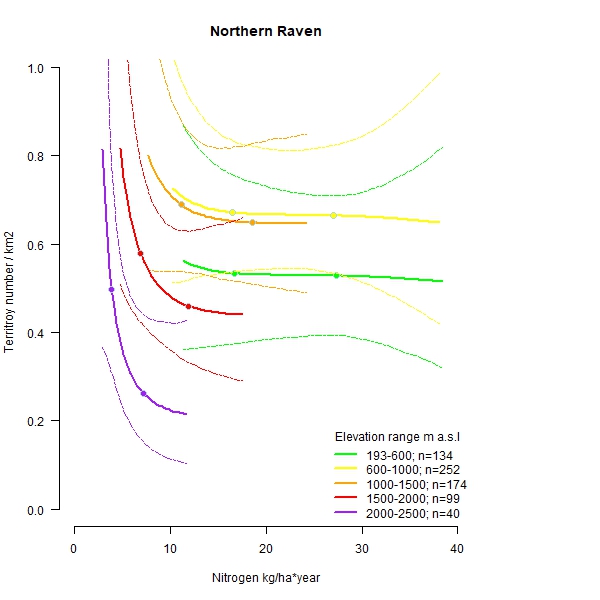

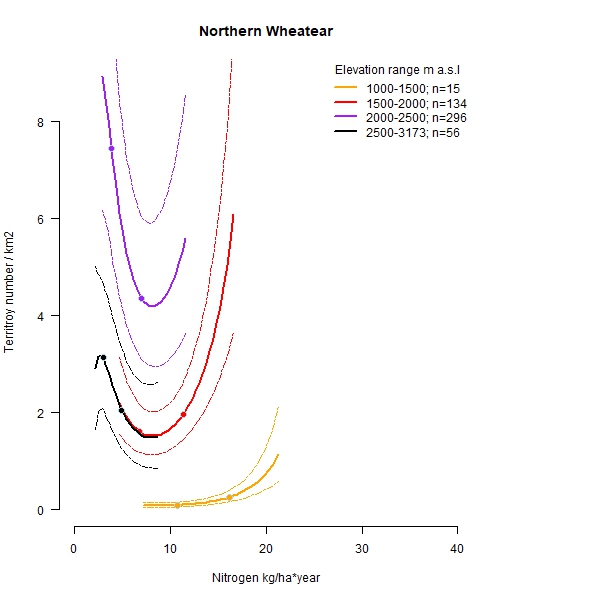

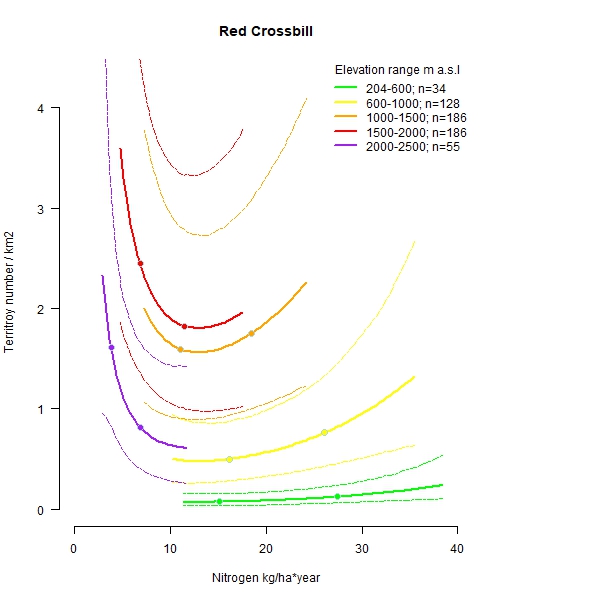

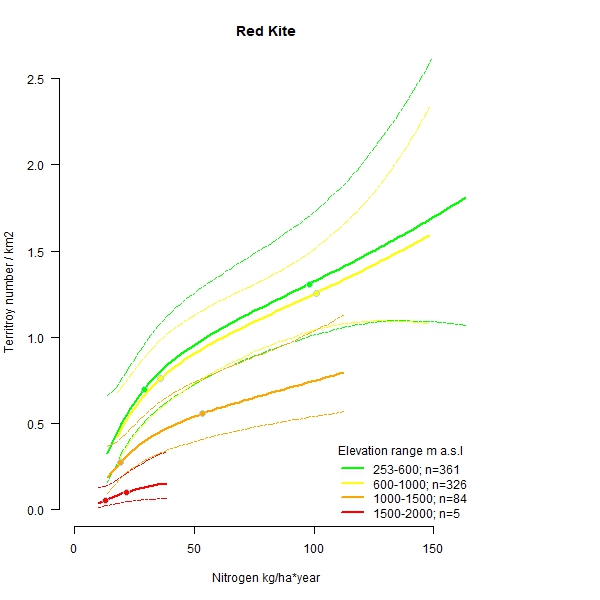

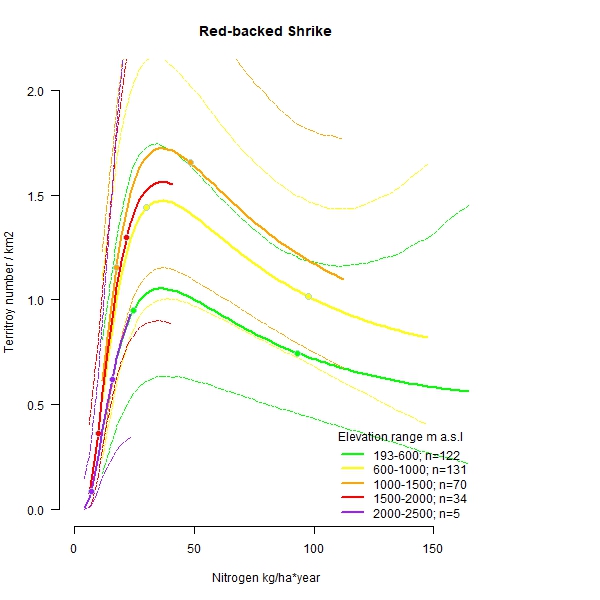

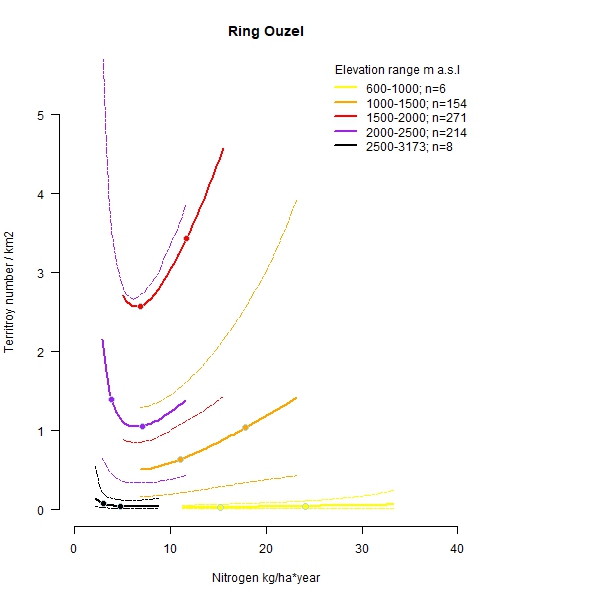

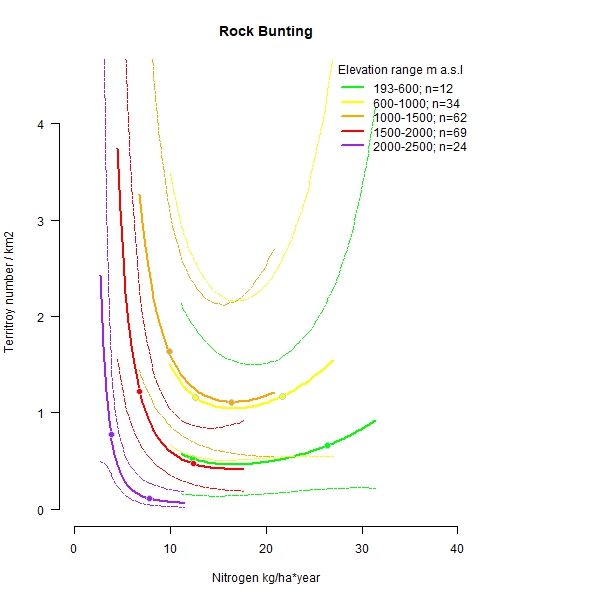

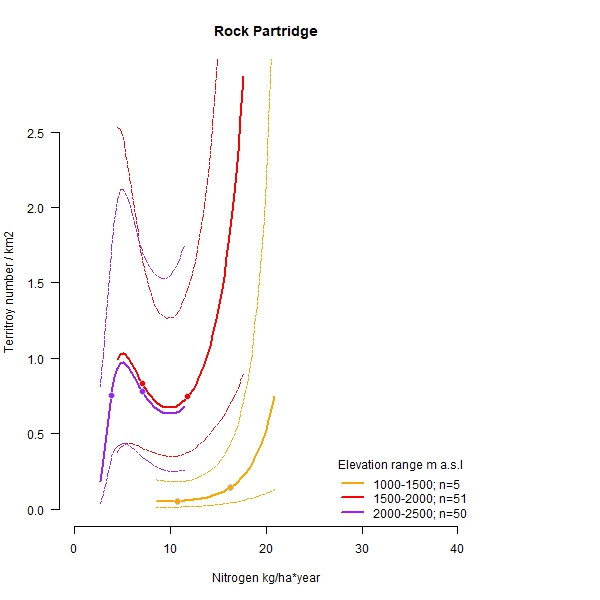

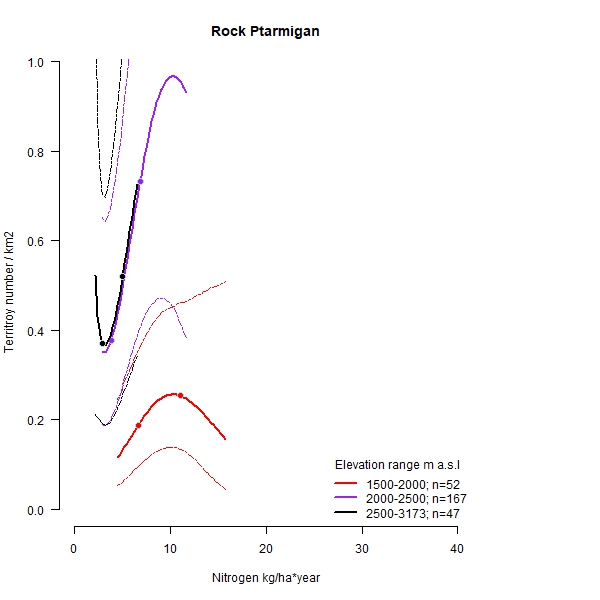

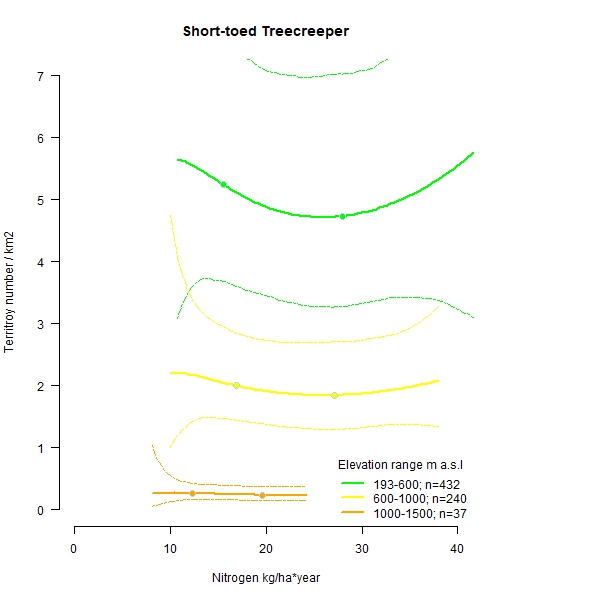

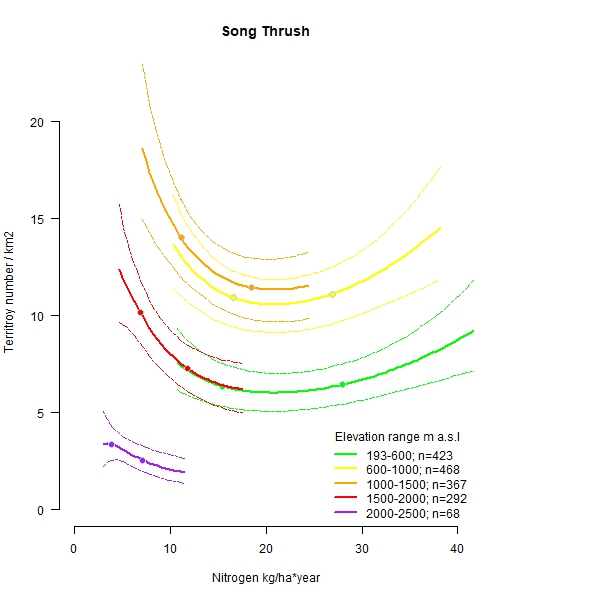

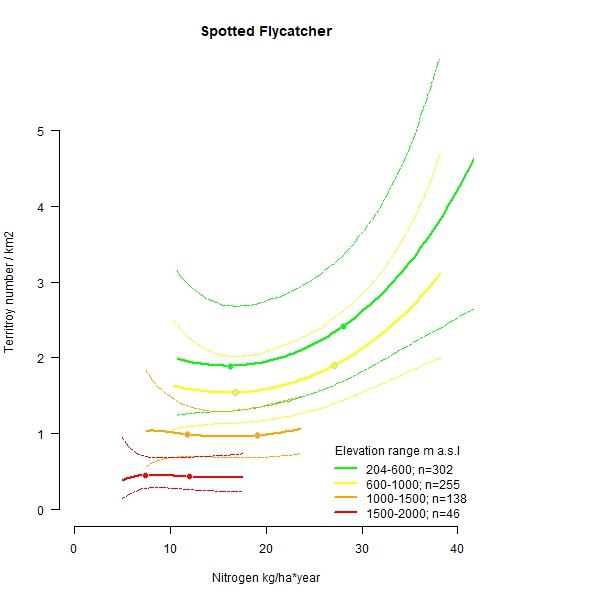

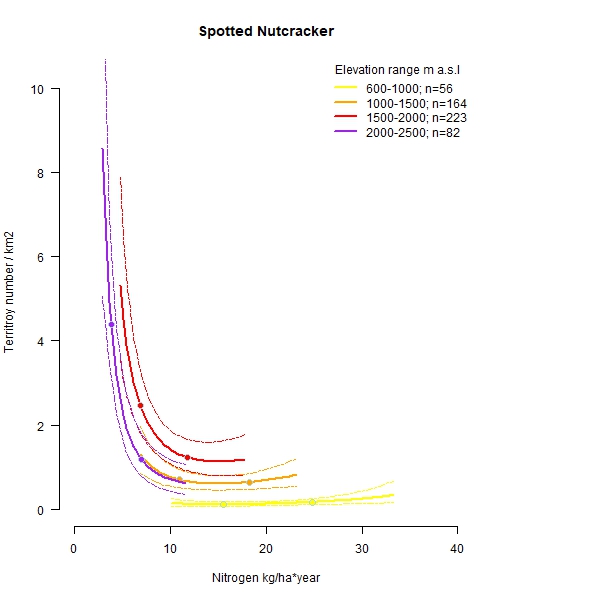

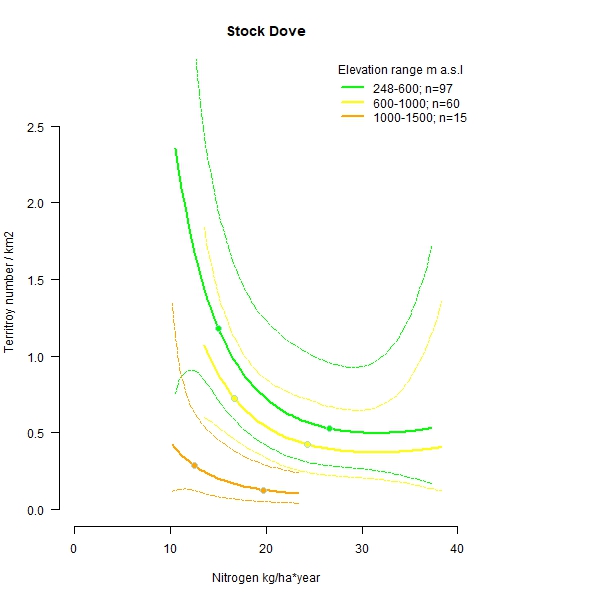

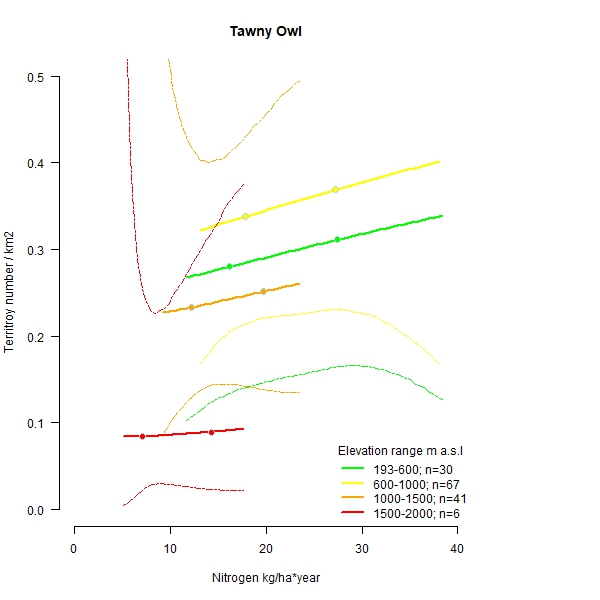

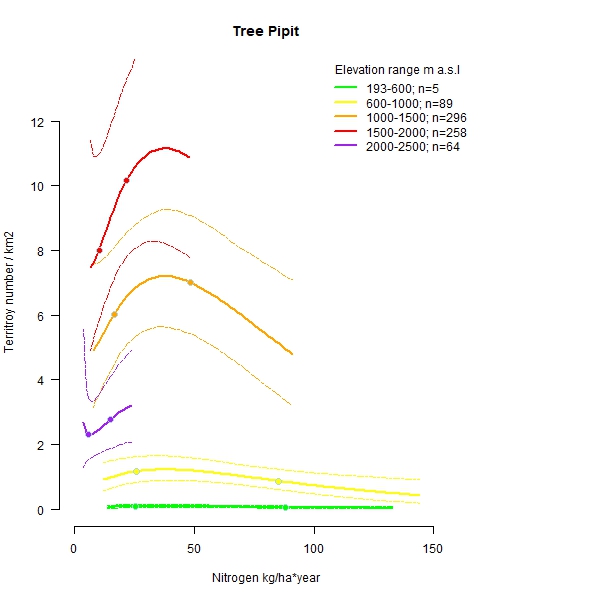

Supplement: Supplementary file 3 — Supporting Information [file COBI-39-e70114-s001.docx]
